# The Wnt receptor Frizzled3 (FZD3) drives aggressive phenotypes in small cell lung cancer

**DOI:** 10.1186/s12931-026-03634-1

**Published:** 2026-03-21

**Authors:** Mingjun Lu, Xiaoyue Zhu, Chenyang Wang, Jiabao Hou, Jingwei Guo, Jiaqi Zhao, Zhendong Qin, Jinghong Wu, Xiaoqing Cao, Tongmei Zhang, Dongchang Wang, Teng Ma

**Affiliations:** 1 Cancer Research Center, Beijing Chest Hospital, Capital Medical University, Beijing Tuberculosis and Thoracic Tumor Research Institute, Beijing, 101149 China; 2 Department of Thoracic Surgery, Beijing Chest Hospital, Capital Medical University, Beijing, 101149 China; 3 Department of Oncology, Beijing Chest Hospital, Capital Medical University, Beijing, 101149 China; 4 Department of Respiratory and Critical Care Medicine, Beijing Chest Hospital, Capital Medical University, Beijing, 101149 China

**Keywords:** FZD3, Small cell lung cancer (SCLC), Wnt signaling pathway, Machine learning

## Abstract

**Background:**

Small cell lung cancer (SCLC) is an aggressive neuroendocrine malignancy characterized by rapid progression and poor prognosis. This study integrates bioinformatics with experimental validation to characterize the role of Frizzled-3 (FZD3), a Wnt receptor, in SCLC progression.

**Methods:**

We analyzed transcriptomic data from 102 SCLC and 55 normal lung tissues retrieved from the Gene Expression Omnibus (datasets GSE6044, GSE40275, and GSE60052). Differential expression analysis was performed using the limma package, followed by GO and KEGG pathway enrichment analyses. To screen for robust prognostic markers, we employed machine learning algorithms—specifically LASSO and Random Forest—to select hub genes. The prognostic significance of FZD3 was assessed using multivariate Cox regression and Kaplan-Meier survival analysis. Validation assays, including qRT-PCR, Western blotting, and functional assays (proliferation, migration, invasion, and apoptosis), were conducted in SCLC cell lines and clinical specimens.

**Results:**

A total of 1,192 differentially expressed genes were identified. Enrichment analysis revealed significant involvement in immune-related pathways and Wnt signaling. FZD3 was selected as a key hub gene and found to be upregulated in SCLC tissues and cell lines. High FZD3 expression was correlated with advanced clinical stage(by Kruskal-Wallis test), and poor prognosis (by Survival analysis). In vitro function assays demonstrated that FZD3 knockdown significantly attenuated SCLC cell proliferation, migration, and invasion while inducing apoptosis.

**Conclusion:**

FZD3 is frequently overexpressed in SCLC and serves as an independent prognostic indicator for poor survival. Our findings elucidate the oncogenic role of FZD3 in SCLC, highlighting its potential as a therapeutic target and prognostic biomarker.

**Supplementary Information:**

The online version contains supplementary material available at 10.1186/s12931-026-03634-1.

## Introduction

Lung cancer remains the leading cause of cancer-related mortality worldwide, with projections indicating it will sustain this burden through 2025 [1, 2]. Among its histological subtypes, small cell lung cancer (SCLC) represents an exceptionally aggressive neuroendocrine malignancy, accounting for 10%–15% of cases [3]. Although tobacco exposure is the dominant risk factor, the molecular mechanisms driving SCLC tumorigenesis remain poorly characterized [4, 5]. While targeted therapies and immunotherapies have revolutionized outcomes for non-small cell lung cancer (NSCLC) over the past decade [6, 7]. SCLC treatment paradigms have largely stagnated. Characterized by early systemic metastasis, rapid progression, and inevitable acquisition of chemoresistance, SCLC patients face a dismal prognosis, with a five-year survival rate of approximately 7% [8–10].

The genomic landscape of SCLC has been difficult to map, primarily due to its exclusion from large-scale sequencing initiatives like TCGA or PCAWG. Nevertheless, available data reveal a genome defined by profound instability, near-universal inactivation of the tumor suppressors *TP53* and *RB1*, and a high tumor mutational burden. These hallmark alterations result in a defective G1/S checkpoint, rendering SCLC cells heavily reliant on G2/M checkpoint mechanisms for DNA repair [11]. Consequently, regulators of this checkpoint, such as ZC3H12A and WEE1, are increasingly viewed as potential therapeutic vulnerabilities [12, 13].

Clinically, treatment stratification depends on staging: limited-stage (LS-SCLC), confined to the hemithorax, and extensive-stage (ES-SCLC), which has spread distally. Due to the metastatic nature of the disease, surgical intervention is rarely feasible, and over 70% of patients present with ES-SCLC [14]. For decades, the standard of care relied exclusively on platinum-based chemotherapy. Although SCLC exhibits a high initial response rate, relapse typically occurs within months due to emergent resistance [15, 16]. A paradigm shift occurred in 2018 with the integration of anti-PD-L1 inhibitors (atezolizumab or durvalumab) into first-line chemotherapy, which significantly improved survival in treatment-naïve ES-SCLC patients. However, long-term benefits are limited to a small subset of patients [17].

Although new immunotherapy targets for SCLC are currently under development, and the first human CAR-T cell trials for SCLC are underway [18–21], there is still an urgent need to identify novel biomarkers that can guide precision medicine and overcome resistance.

Integrated bioinformatics and machine learning offer a robust framework to decode the complex molecular networks of SCLC and identify reliable therapeutic targets [22–24]. In this study, we leveraged a dual machine-learning approach to screen for hub genes within SCLC transcriptomic datasets, identifying Frizzled-3 (FZD3) as a critical candidate. We further validated the prognostic value of FZD3 in clinical cohorts and mechanistically characterized its role in driving aggressive SCLC phenotypes in vitro. This integrative strategy highlights FZD3 as a potential therapeutic target to improve patient outcomes.

## Materials and methods

### Data collection and processing

Datasets comprising 102 SCLC samples and 55 normal samples were downloaded from the GEO database under accession numbers GSE6044, GSE40275, and GSE60052. To assess overall variability and group separation among samples, principal component analysis (PCA) was performed on the gene expression data [25]. First, gene expression values from all samples were extracted and log-transformed to obtain FPKM-normalized data. The preprocessed expression matrix was then subjected to PCA using the prcomp function. The first two principal components (PC1 and PC2), which typically account for the majority of variance in the dataset, were selected for visualization. Finally, the ggplot2 package was used to generate PCA plots illustrating sample distribution.

### Identification of differentially expressed genes (DEGs)

The limma package [26] was employed to identify DEGs between SCLC patients and normal controls. DEGs were defined as those with |log₂FoldChange| > 1 and an adjusted p-value < 0.05. The p-values were adjusted for multiple testing using the Benjamini-Hochberg (BH) method. To visualize the distribution of DEGs, volcano plots were generated using the ggplot2 package, and a heatmap of the top 50 most significantly altered genes was plotted using the pheatmap package.

### Functional enrichment analysis of DEGs

To explore the biological functions and pathways associated with the DEGs, Gene Ontology (GO) and Kyoto Encyclopedia of Genes and Genomes (KEGG) pathway analyses were conducted using the clusterProfiler package [27]. GO analysis covered three categories: Cellular Component (CC), Biological Process (BP), and Molecular Function (MF). Significantly enriched terms and pathways were selected based on a p-value < 0.05. Results were visualized using the ggplot2 package.

### Machine learning-based identification of hub genes

Genes involved in the top ten enriched KEGG pathways and GO terms were further analyzed using two machine learning algorithms to identify hub genes. First, to select genes with the strongest discriminatory power between SCLC and normal tissues, we employed Least Absolute Shrinkage and Selection Operator (LASSO) regression, which was implemented via the glmnet package, the LASSO model was fitted using a binary response variable (1 for SCLC, 0 for Normal) with family = “binomial” and alpha = 1 for LASSO regularization, to select the most relevant features from high-dimensional data [28]. Additionally, the random forest algorithm was applied using the randomForest package. Gene importance was measured by the Mean Decrease in Gini index, and genes with an importance score > 2 were selected. The final set of hub genes was determined by taking the intersection of genes selected by both methods using the venn() function.

### Multivariate cox proportional hazards regression

To identify independent prognostic factors, multivariate Cox regression analysis was performed using the survival R package to evaluate variables significantly associated with patient survival time and status. Variables selected from both LASSO and random forest analyses were included in the multivariate model. Results are presented as hazard ratios (HR) with 95% confidence intervals (CI).

### Survival analysis of FZD3

Clinical information corresponding to each dataset was downloaded, and samples with incomplete survival time or status were excluded. The predict() function was used to calculate a risk score for each patient, with the median risk score serving as the cutoff to classify patients into high- and low-risk groups. Kaplan–Meier survival analysis was then conducted using the survival package in R to compare survival differences between the two groups.

### Clinical sample collection

SCLC tumor tissues and adjacent normal tissues were obtained from the Biobank of Beijing Chest Hospital. Adjacent tissues were collected at least 5 cm away from the tumor margin. Tissues were paraffin-embedded after resection. This study was approved by Beijing Chest Hospital and conducted in accordance with the Declaration of Helsinki and Good Clinical Practice guidelines, and the study protocol was reviewed and approved by the Ethics Committee of Beijing Chest Hospital Affiliated to Capital Medical University.

### mIHC

The PANO IHC Kit (Panovue, Beijing, China, 0003100100) was employed, with the operational procedure strictly adhering to the manufacturer’s instructions. SCLC tumor tissues (*n* = 80) and adjacent normal tissues (*n* = 80) sections underwent dewaxing followed by antigen retrieval, after which they were stained using antibodies as follows: FZD3 1:1500 (Immunoway, #YT1777). The stained slides were scanned using a Vectra multispectral microscope (Akoya Biosciences, RRID: SCR_023774). Monochromatic images of the stained tissue sections were acquired, and the spectra of each fluorophore were extracted to create the required spectral library for multiplex immunohistochemical staining, which was performed in InForm (v2.6.0, Akoya Biosciences).

### Cell culture and transfection

SCLC cell lines (DMS 114, NCI-H446) were purchased from Procell (Wuhan, China) and cultured in complete medium (RPMI-1640 supplemented with 10% FBS, 100 µg/mL penicillin, and 100 µg/mL streptomycin) at 37 °C under 5% CO₂. Small interfering RNA targeting FZD3 (FZD3-siRNA) and negative control siRNA (NC-siRNA) were synthesized by RiboBio (Guangzhou, China). Cells were transfected using Lipofectamine^®^ RNAiMAX (Thermo Fisher, #13778-150) according to the manufacturer’s instructions and incubated for 48 h prior to subsequent experiments. Transfection conditions were scaled proportionally based on cell number and plate format.

### RNA extraction and quantitative real-time PCR (qRT-PCR)

Total RNA was extracted using TRIzol™ Reagent (Thermo Fisher, #15596018CN). cDNA was synthesized from 1 µg of total RNA using HiScript IV All-in-One Ultra RT SuperMix (Vazyme, #R433-01) following the manufacturer’s protocol. qRT-PCR was performed using Taq Pro Universal SYBR qPCR Master Mix (Vazyme, #Q712-02) on an Applied Biosystems 7500 Real-Time PCR System. β-actin was used as the internal control. Relative gene expression was calculated using the 2^(-ΔΔCt) method.

Primer sequences:

- β-actin-Fw: CCTGGCACCCAGCACAAT.
- β-actin-Rv: GGGCCGGACTCGTCATAC.
- FZD3-Fw: TGGAATGCAGTAGGTTCCCA.
- FZD3-Rv: TCGGGGACACCAAAAACCAT.

### Western blotting

Proteins were extracted using RIPA buffer containing protease inhibitors (Solarbio, #R0010). Protein lysates (5 µg per sample) were separated by 10% SDS-PAGE and transferred to nitrocellulose (NC) membranes. Membranes were blocked with 5% skim milk in TBST for 1 h and then incubated overnight at 4 °C with an anti-FZD3 primary antibody (1:2000; Immunoway, #YT1777). After washing with TBST, membranes were incubated with an HRP-conjugated anti-rabbit IgG secondary antibody (1:10000; Proteintech, #SA00001-2) for 1 h at room temperature. GAPDH (Proteintech, #HRP-60004) served as the loading control. Blots were developed using an ultrasensitive chemiluminescence kit (NCM Biotech, #P10300) and quantified with ImageJ software.

### Immunofluorescence

One hundred thousand cells per well were seeded in a 12-well plate, then transfect with siRNA for 48 h. Cells were fixed with 4% paraformaldehyde for 15 min, washed three times with PBS, permeabilized with 0.3% Tween-20 for 10 min, and blocked with 10% fetal bovine serum for 45 min. The cells were then incubated with primary antibody anti-β-catenin 1:400 (Immunoway, #YM8174) at room temperature for 2 h, followed by three washes with PBS. Next, the cells were incubated with secondary antibody at room temperature for 1 h, and washed three times with PBS. Finally, the samples were mounted with DAPI and examined under a confocal microscope.

### Colony formation assay

SCLC cells (DMS 114, NCI-H446) were transfected with siRNA for 24 h, counted, and seeded at 3000 cells per well in 6-well plates. After 14 days of culture at 37 °C and 5% CO₂, cells were fixed with 4% paraformaldehyde for 15 min and stained with crystal violet (Beyotime, #C0121) for 1 h. Plates were rinsed with deionized water, air-dried, and imaged. Colonies were quantified using ImageJ.

### CCK-8 proliferation assay

Cells were transfected with siRNA for 24 h, counted, and plated at 1000 cells per well in 96-well plates. After 24 h of culture, 200 µL of CCK-8 reagent (Gipbio, #GK10001) diluted 1:100 in serum-free medium was added to each well. Plates were incubated for 2 h at 37 °C under 5% CO₂, and absorbance at 450 nm was measured. Measurements were taken at 24, 48, 72, and 96 h following the same protocol.

### Migration and invasion assays

#### Migration

After 24 h of siRNA transfection, cells were suspended in serum-free RPMI-1640 medium, counted, and seeded into 24-well Transwell plates (Corning, #WG3422) at 3 × 10⁴ cells per well. After 24 h, cells were fixed with 4% paraformaldehyde, stained with crystal violet, and imaged.

#### Invasion

Cells were transfected as above and seeded into Matrigel-coated (Corning, #354234) Transwell inserts at 3 × 10⁴ cells per well. After 24 h, invaded cells were fixed, stained, and counted.

### Apoptosis and cell cycle analysis by flow cytometry

#### Apoptosis

Cells in the logarithmic growth phase were seeded in 6-well plates and transfected with siRNA for 48 h. Apoptosis was detected using an Annexin V-FITC/PI Apoptosis Kit (Elabscience, #ECKA211) according to the manufacturer’s instructions. Data were acquired on a flow cytometer and analyzed with FlowJo software.

#### Cell cycle

After transfection, cells were fixed in 70% ethanol at 4 °C for ≥ 30 min, stained with PI using a Cell Cycle Kit (Beyotime, #C1052), and analyzed by flow cytometry.

## Results

### Identification of differentially expressed genes in tumor and normal samples

**Fig. 1:**
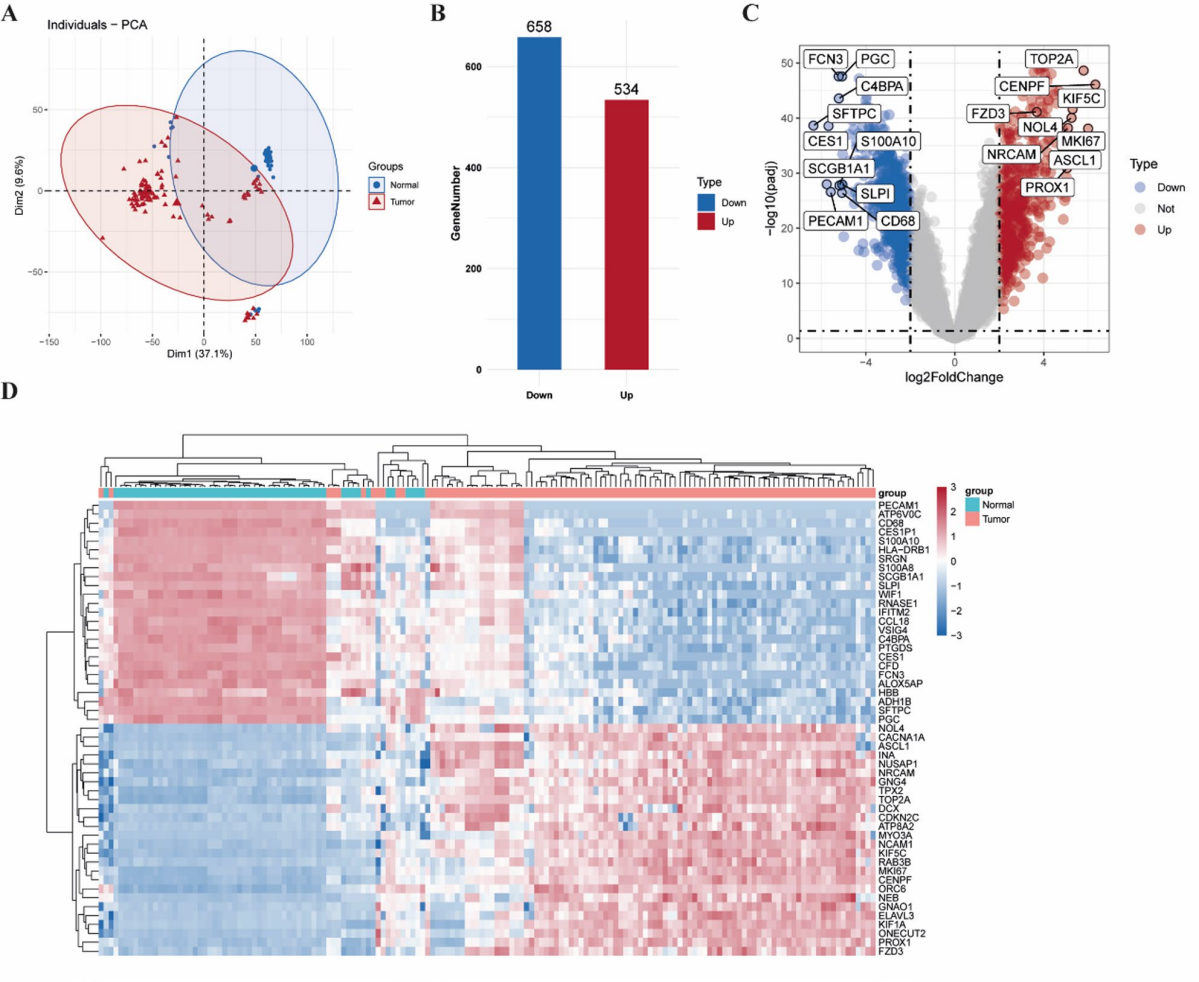
Identification of differentially expressed genes (DEGs) between SCLC patients and normal controls. **A** Principal component analysis (PCA) plot illustrating the overall distribution of samples based on gene expression profiles. **B** Bar graph displaying the number of up-regulated and down-regulated DEGs. **C** Volcano plot of the DEGs. Red dots represent significantly up-regulated genes, and blue dots represent significantly down-regulated genes. **D** Heatmap of the top 100 DEGs, comparing the expression patterns between the tumor group and the normal control group

To investigate differentially expressed genes (DEGs) between SCLC tissues and normal lung tissues, we downloaded gene expression datasets GSE6044, GSE40275, and GSE60052 from the GEO database, comprising a total of 102 SCLC samples and 55 normal lung tissue samples. After preprocessing and data cleaning, the expression matrices were normalized, and the effectiveness of normalization was verified by PCA (Fig. 1A). Subsequently, differential expression analysis was performed using the “limma” R package, identifying 1192 DEGs, including 658 downregulated and 534 upregulated genes (Fig. 1B–C). The top 100 DEGs ranked by absolute logFC values are displayed in a heatmap (Fig. 1D), and detailed information of all DEGs is provided in Supplementary Table S1.

### Functional enrichment analysis of DEGs via GO and KEGG

**Fig. 2:**
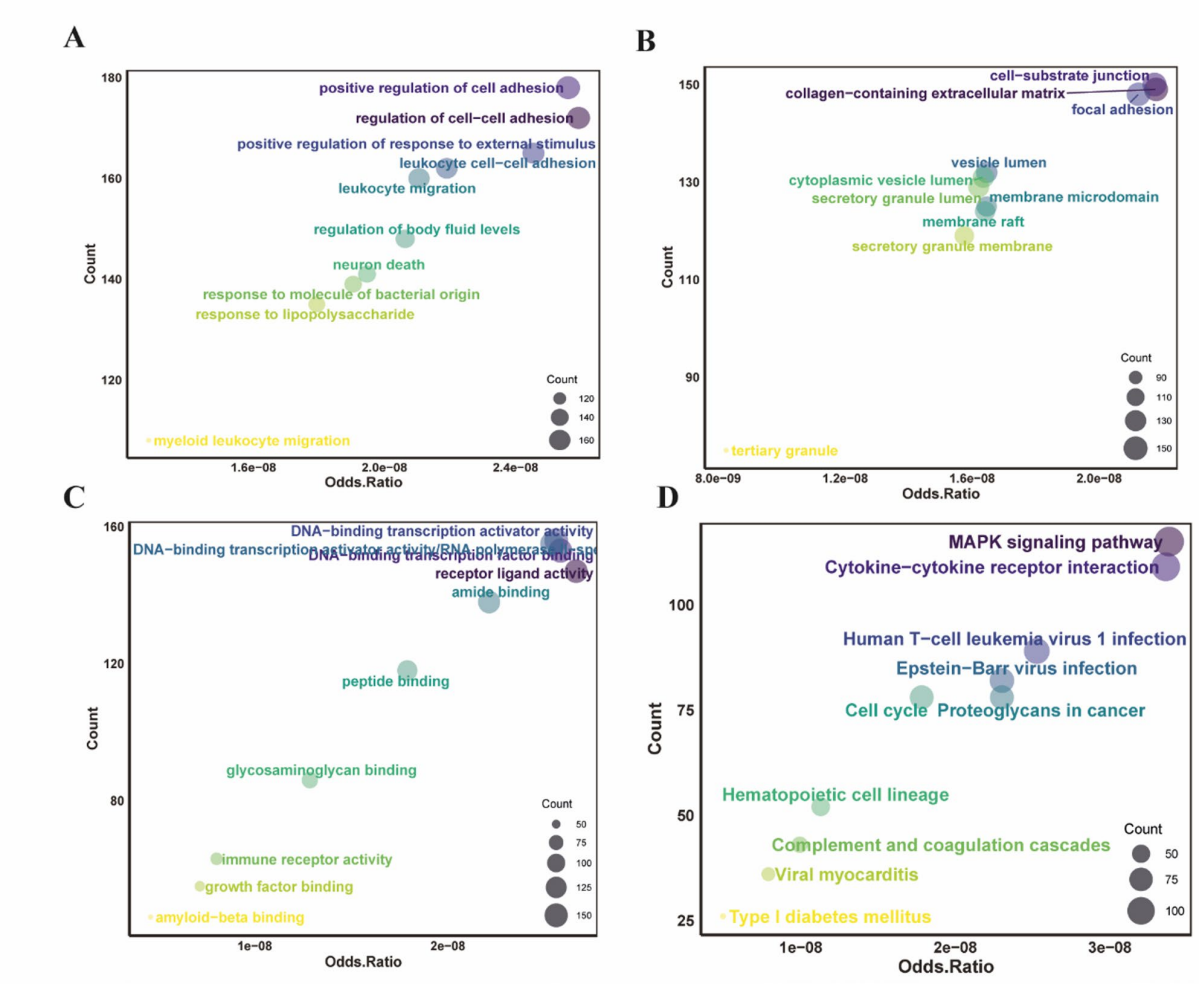
Gene Ontology (GO) and KEGG enrichment functional analysis of the differentially expressed genes. **A** Top ten significantly enriched terms in GO Biological Process (BP). **B** Top ten significantly enriched terms in GO Cellular Component (CC). **C** Top ten significantly enriched terms in GO Molecular Function (MF). **D** Top ten significantly enriched pathways in the KEGG analysis

To elucidate the key biological functions of the identified DEGs in SCLC, GO and KEGG enrichment analyses were conducted. The GO analysis covered three categories: BP, CC, and MF. The KEGG analysis focused on pathways related to metabolism, genetic information processing, environmental information processing, cellular processes, organismal systems, and human diseases. The results revealed that in BP, DEGs were significantly enriched in processes such as leukocyte migration, leukocyte cell-cell adhesion, positive regulation of cell adhesion, and myeloid leukocyte migration (Fig. 2A). In CC, DEGs were primarily associated with structures involved in cell signaling and membrane transport, including vesicle lumen, cytoplasmic vesicle lumen, and membrane microdomain (Fig. 2B). For MF, significant enrichment was observed in DNA-binding transcription activator activity RNA polymerase II-specific, peptide binding, and amyloid-beta binding (Fig. 2C). KEGG analysis further indicated that these genes play important roles in pathways such as the MAPK signaling pathway, cytokine-cytokine receptor interaction, and human T-cell leukemia virus 1 infection (Fig. 2D).

### Identification of the key gene FZD3

**Fig. 3:**
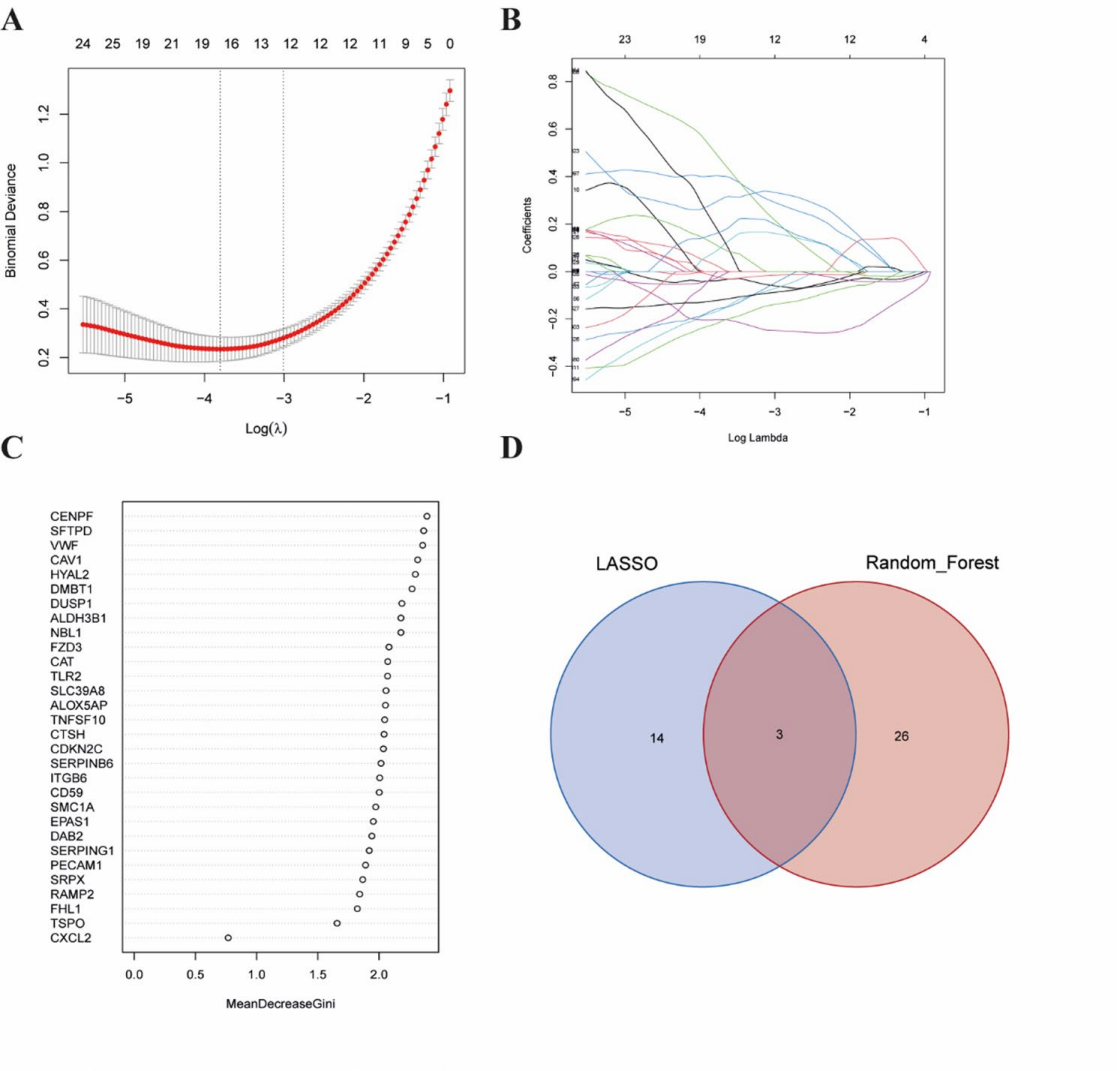
Screening of hub genes using two machine learning algorithms. **A** Coefficient profile plot from the LASSO regression model showing the trajectories of each coefficient against the log(λ) values. **B** Genes selected by the LASSO regression model with non-zero coefficients. **C** Ranking plot of gene importance scores based on the random forest algorithm. **D** Venn diagram identifying the hub genes commonly recognized by the two algorithms

Based on the functional enrichment results, genes involved in the top ten enriched GO terms and KEGG pathways were selected. Two machine learning algorithms, LASSO regression and random forest, were employed to screen for disease-related hub genes (Fig. 3A–C). LASSO analysis identified 17 hub genes, while random forest yielded 29. Through Venn diagram analysis, three overlapping hub genes—EPAS1, FZD3, and SRPX—were identified (Fig. 3D).

**Fig. 4:**
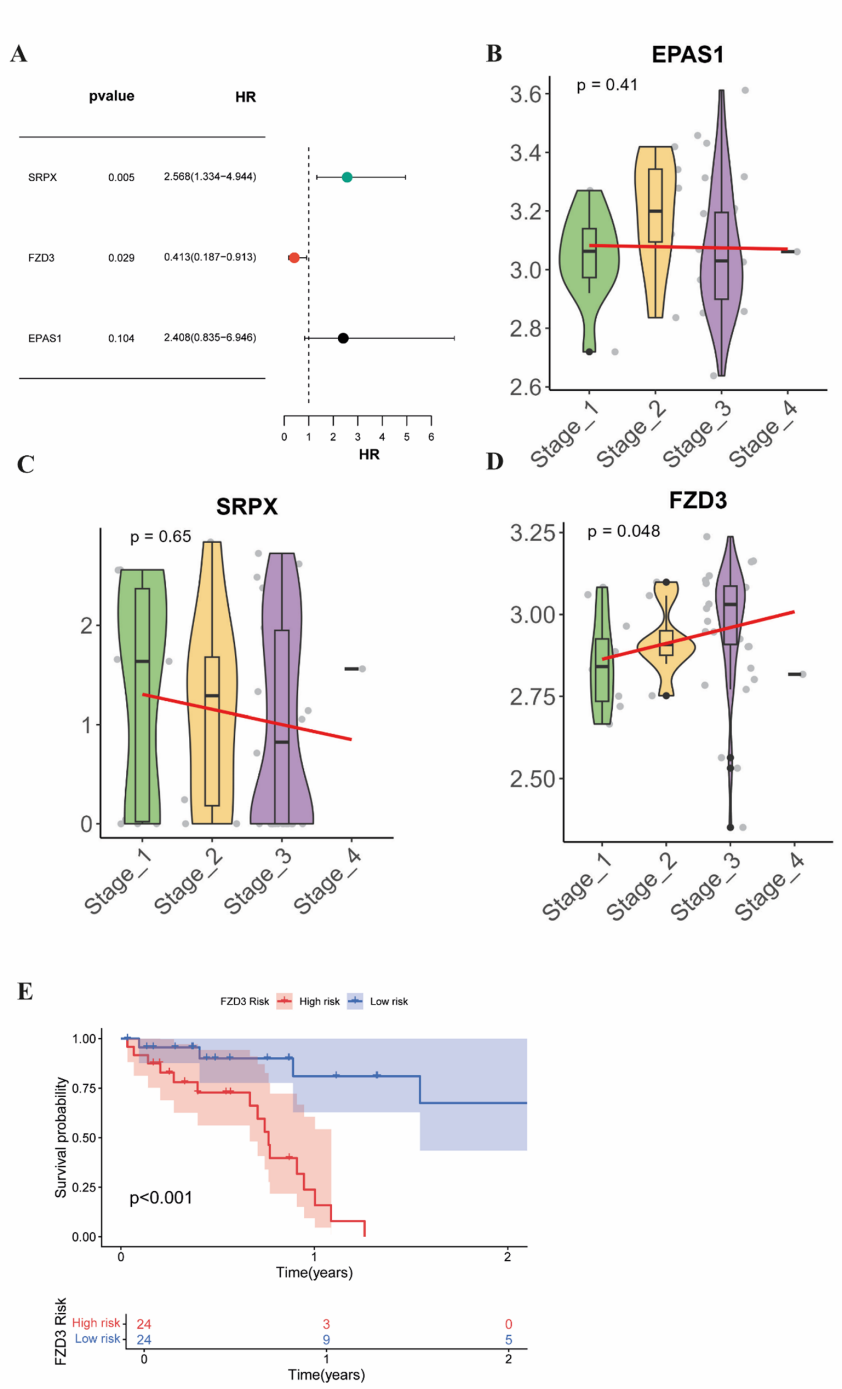
Clinical characteristics and risk factor analysis of FZD3. **A** Forest plot of hazard ratios for the three hub genes. **B-D** Box plots showing the expression levels of EPAS1, SRPX, and FZD3 across different Tumor Stages. **E** Kaplan-Meier survival curve analysis for groups with high and low FZD3 expression

To evaluate the association of EPAS1, FZD3, and SRPX with patient survival, a multivariate Cox regression model was applied. The results showed that only FZD3 exhibited a significant correlation (*P* < 0.05), while the other two genes did not reach significance (Fig. 4A). Further analysis incorporating clinical staging data revealed that only FZD3 expression varied significantly across different stages (Fig. 4B–D). Finally, Kaplan–Meier survival analysis demonstrated that patients with high FZD3 expression had significantly shorter overall survival compared to those with low expression (Fig. 4E), indicating that high FZD3 expression may serve as a potential indicator of poor prognosis in SCLC patients. Based on these findings, FZD3 was selected as the target gene for subsequent investigations.

### Elevated expression of FZD3 in SCLC tissues and cell lines

**Fig. 5:**
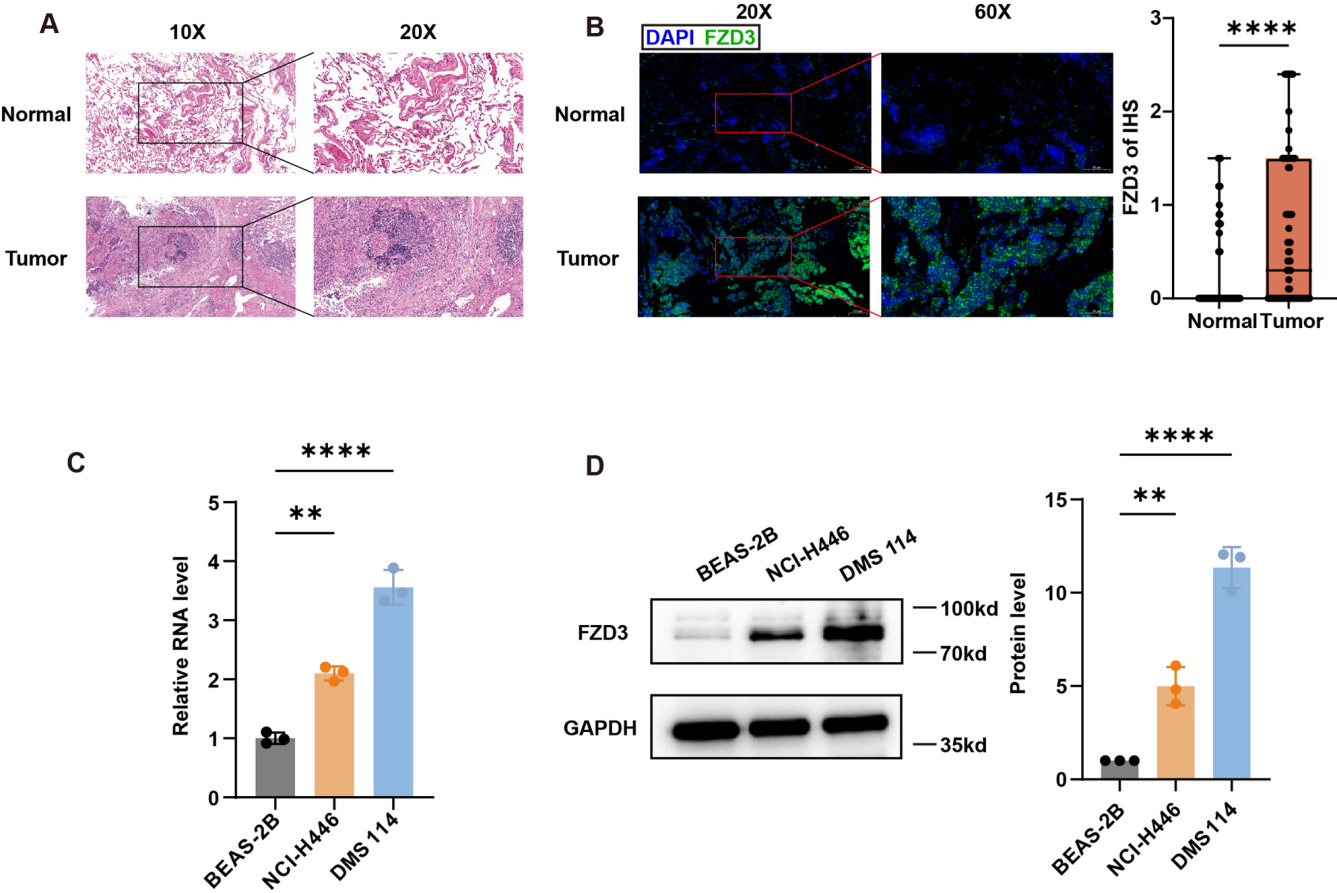
Validation of FZD3 expression in clinical samples and cell lines. **A** HE staining of tumor and adjacent normal tissues from SCLC patients (n = 80). Student’s t test analysis was performed. Error bars represent the standard deviation (SD) from three replicate experiments. **B** Differential expression of FZD3 in tumor versus adjacent normal tissues detected by immunohistochemistry. **C** FZD3 mRNA expression levels in SCLC cell lines (NCI-H114, NCI-H446) and normal bronchial epithelial cells measured by RT-qPCR. **D** FZD3 protein expression in SCLC cell lines (NCI-H114, NCI-H446) and normal bronchial epithelial cells detected by Western blot. One-way ANOVA analysis was performed. Error bars represent the standard deviation (SD) from three replicate experiments. * *p* < 0.05 **p < 0.01, ** *p* < 0.001, **** *p* < 0.0001

To evaluate FZD3 expression patterns in SCLC, we analyzed 80 SCLC tumor specimens alongside adjacent normal tissues (clinical characteristics detailed in Table S2). Histological confirmation via hematoxylin and eosin (H&E) staining (Fig. 5A) was followed by multiplex immunohistochemical (mIHC) analysis. FZD3 localized predominantly to the cell membrane, showing a significantly higher positive rate in SCLC tissues compared to normal controls (Fig. 5B). Consistent with tissue data, both mRNA and protein levels of FZD3 were markedly elevated in SCLC cell lines (NCI-H114, NCI-H446) relative to the human normal lung epithelial cell line BEAS-2B (Fig. 5C–D).

### FZD3 silencing suppresses malignant phenotypes and induces apoptosis

**Fig. 6:**
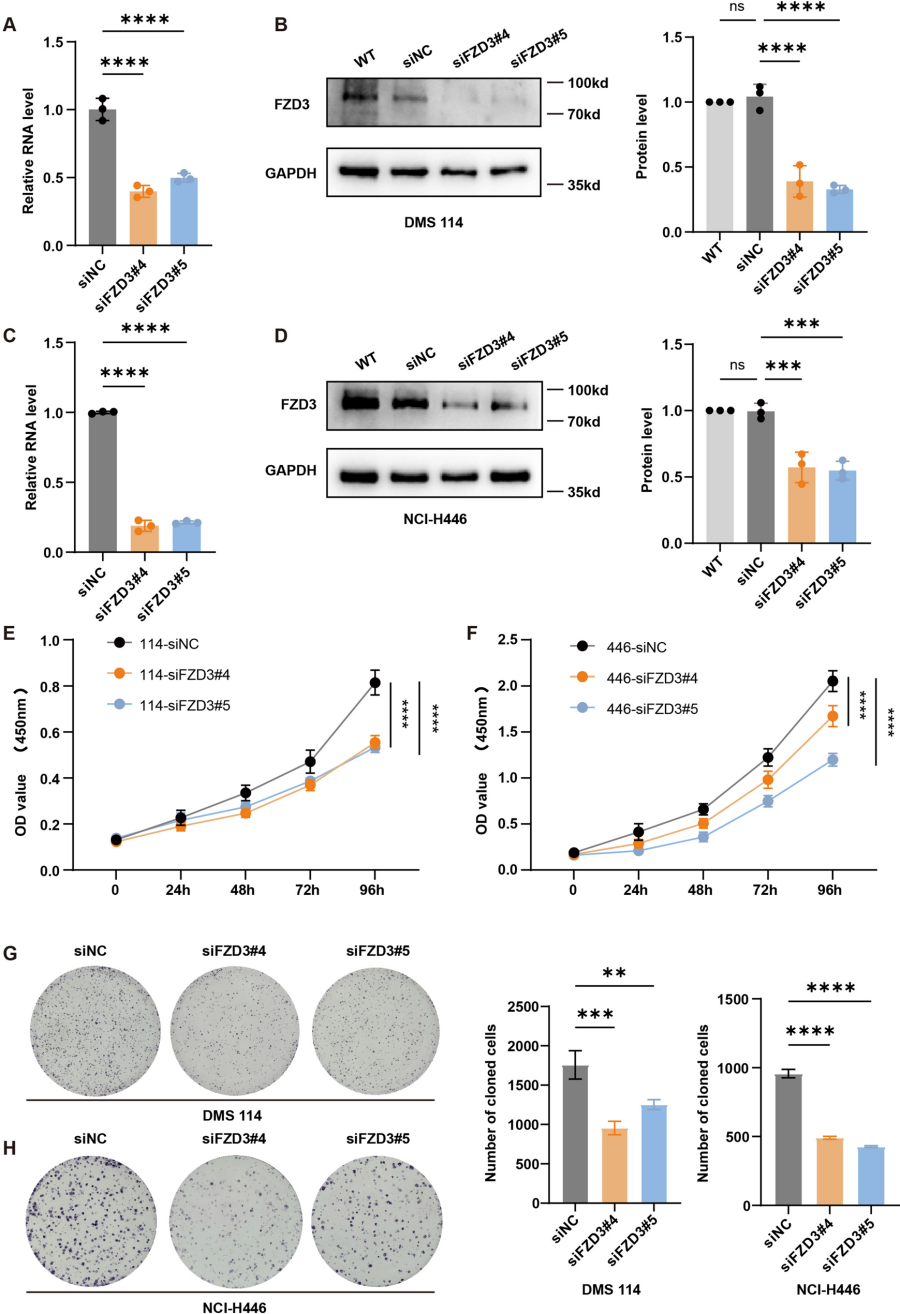
Effect of FZD3 knockdown on SCLC cell proliferation. **A** -**D** Knockdown efficiency of FZD3 in SCLC cell lines evaluated by RT-qPCR and Western blot. One-way ANOVA analysis was performed. Error bars represent the standard deviation (SD) from three replicate experiments. **E** and **F** Proliferation of SCLC cells after FZD3 knockdown (0–96 h) assessed by CCK-8 assay. Two-way ANOVA analysis was performed. Error bars represent the standard deviation (SD) from three replicate experiments. **G** and **H** Colony formation ability of SCLC cells after FZD3 knockdown (14 days) examined by colony formation assay. One-way ANOVA analysis was performed. Error bars represent the standard deviation (SD) from three replicate experiments. * *p* < 0.05 **p < 0.01, ** *p* < 0.001, **** *p* < 0.0001

**Fig. 7:**
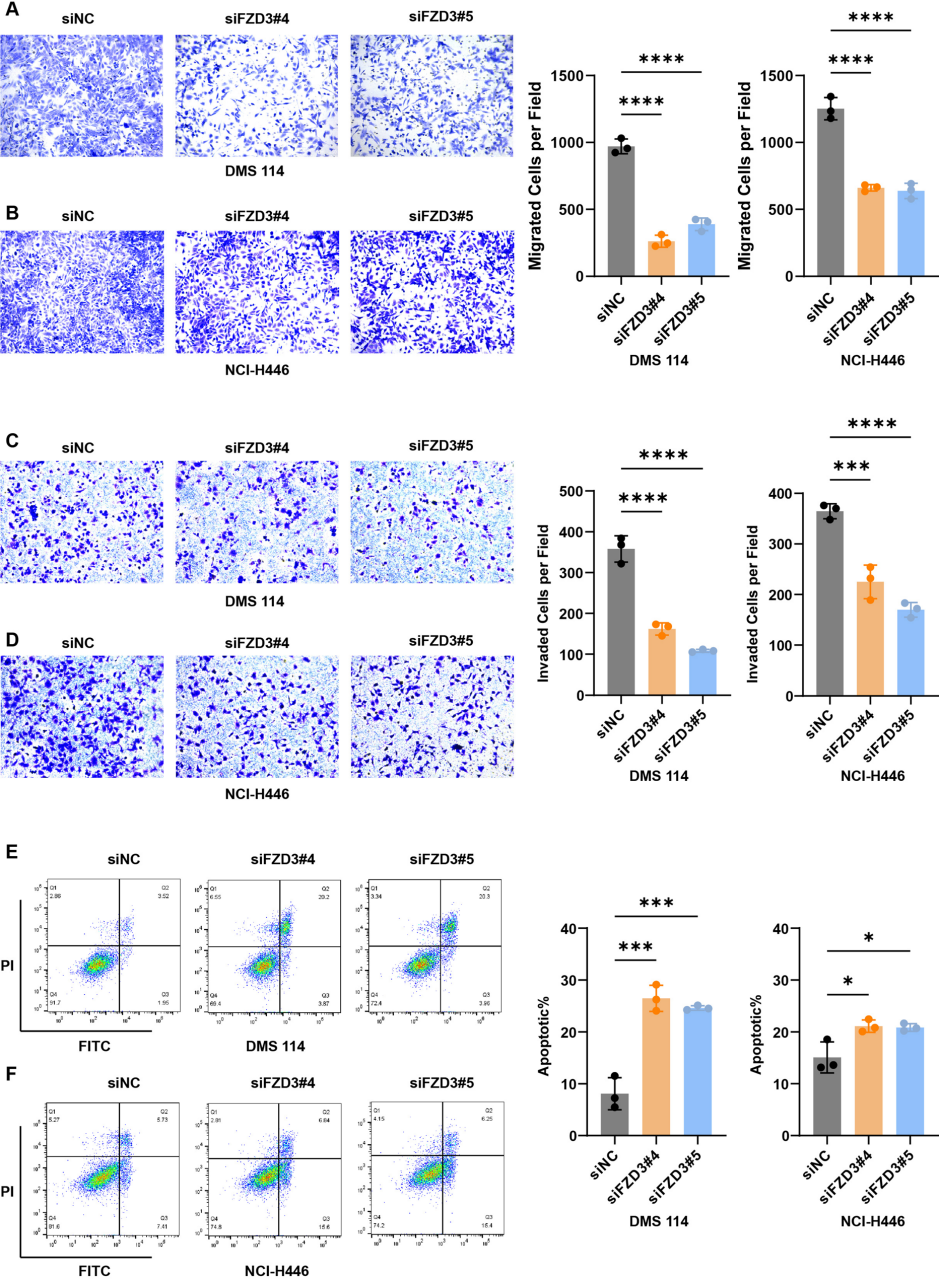
Effect of FZD3 knockdown on migration, invasion, and apoptosis of SCLC cells. **A** and **B** Migration ability of SCLC cells after FZD3 knockdown evaluated by Transwell assay. One-way ANOVA analysis was performed. Error bars represent the standard deviation (SD) from three replicate experiments. **C** and **D** Invasion ability of SCLC cells after FZD3 knockdown evaluated by Transwell assay. One-way ANOVA analysis was performed. Error bars represent the standard deviation (SD) from three replicate experiments. **E** and **F** Apoptosis level of SCLC cells induced by FZD3 knockdown detected by flow cytometry. One-way ANOVA analysis was performed. Error bars represent the standard deviation (SD) from three replicate experiments. * *p* < 0.05 **p < 0.01, ** *p* < 0.001, **** *p* < 0.0001

To assess the oncogenic function of FZD3, we silenced its expression in DMS 114 and NCI-H446 cells using two specific siRNAs. qRT-PCR and Western blotting confirmed robust knockdown efficacy (Fig. 6A–D). Functionally, FZD3 depletion significantly impaired cell viability and clonogenic capacity, as evidenced by CCK-8 and colony formation assays (Fig. 6E–H). Importantly, re-expression of FZD3 rescued these proliferative defects, confirming target specificity (Fig. S2A–C). Similarly, Transwell assays demonstrated that FZD3 knockdown abrogated SCLC cell migration and invasion (Fig. 7A–D), phenotypes that were fully reversible upon FZD3 restoration (Fig. S2D–G). Taken together, these data implicate FZD3 as a critical driver of SCLC growth and metastasis.

To determine the cellular mechanism underlying growth inhibition, we performed cell cycle and apoptosis analyses. FZD3 knockdown did not significantly alter cell cycle distribution (G1/S/G2 phases) (Fig. S1A–B), suggesting that FZD3 modulates SCLC growth via mechanisms distinct from cell cycle arrest. In contrast, Annexin V/PI staining revealed a substantial increase in apoptotic rates in knockdown cells (Fig. 7E–F), indicating that FZD3 promotes SCLC survival primarily by suppressing apoptosis.

### FZD3 drives SCLC aggressiveness via a non-canonical, Wnt-independent axis

**Fig. 8:**
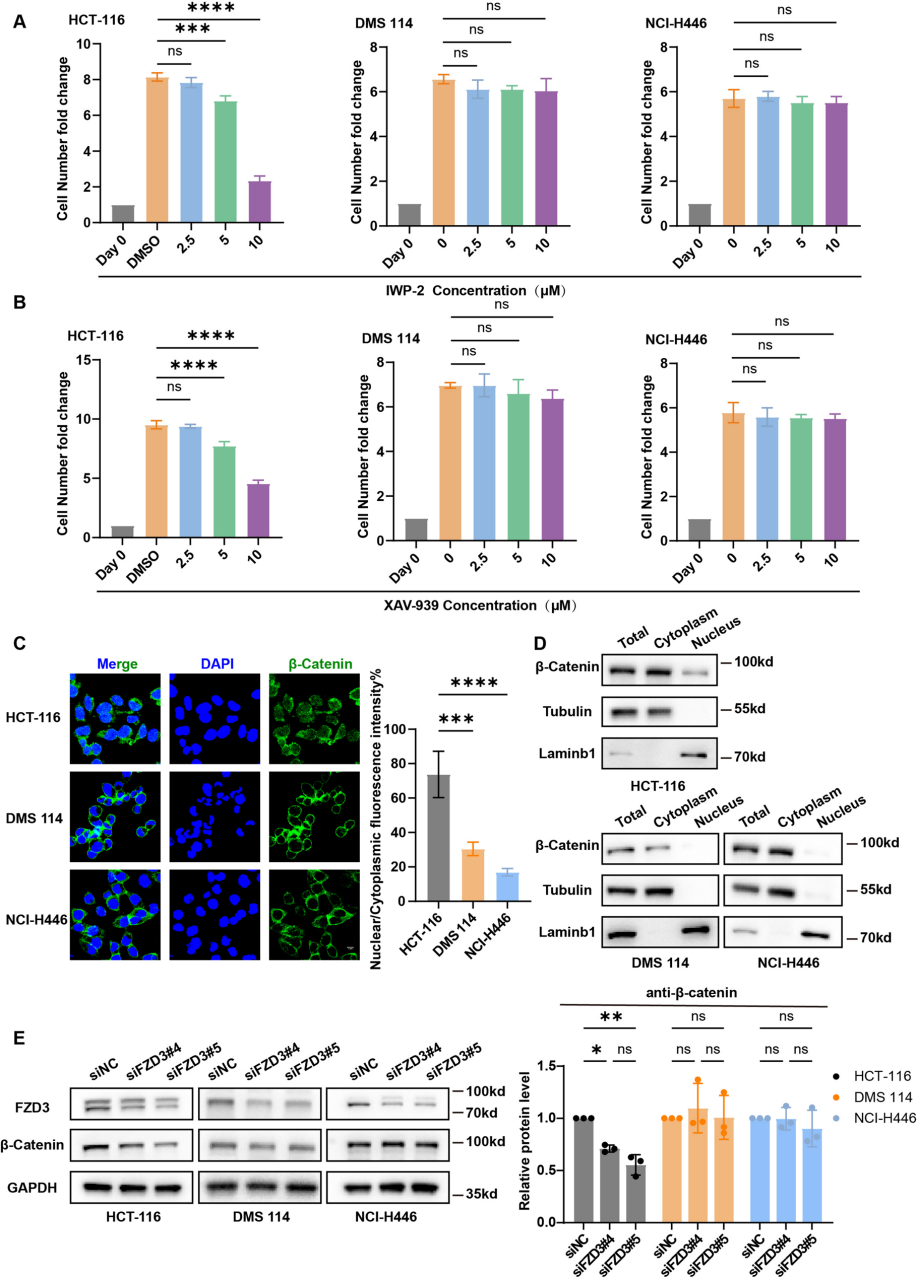
FZD3 regulates small cell lung cancer cells independently of the classical Wnt pathway. **A** and **B** Inhibitors targeting the upstream (IWP2) and downstream (XAV939) components of the WNT pathway were added to assess proliferation in small cell lung cancer cells (NCI-H114, NCI-H446) and colorectal cancer cells (HCT-116). One-way ANOVA analysis was performed. Error bars represent the standard deviation (SD) from three replicate experiments. **C** Immunofluorescence analysis of small cell lung cancer cells and colorectal cancer cells during active growth phase using β-catenin-specific antibodies. Green indicates β-catenin staining positivity. Blue indicates DAPI nuclear staining. **D** Western blot analysis of β-catenin distribution in the cytoplasm and nucleus of small cell lung cancer cells and colorectal cancer cells during active growth phase. **E** Changes in β-catenin protein expression following FZD3 knockdown in small cell lung cancer cells and colorectal cancer cells as detected by Western blot. One-way ANOVA analysis was performed. Error bars represent the standard deviation (SD) from three replicate experiments. * *p* < 0.05 **p < 0.01, ** *p* < 0.001, **** *p* < 0.0001

Given FZD3’s established role as a Wnt receptor, we investigated whether its oncogenic function in SCLC depends on canonical Wnt/β-catenin signaling. We treated SCLC cells with upstream (IWP2) and downstream (XAV939) Wnt inhibitors, using β-catenin-dependent colorectal cancer cells (HCT-116) as a positive control [29]. While both inhibitors suppressed HCT-116 proliferation, they failed to impact SCLC cell growth (Fig. 8A–B), suggesting SCLC insensitivity to canonical Wnt blockade. Mechanistically, immunofluorescence and Western blotting revealed that β-catenin is sequestered in the cytoplasm of SCLC cells, with negligible nuclear translocation required for transcriptional activation (Fig. 8C–E).

To delineate the molecular network driven by FZD3, we performed RNA sequencing (RNA-seq) on DMS 114 and NCI-H446 cells following FZD3 knockdown. Differential expression analysis identified 631 genes (|log₂FC| > 1.0, *P* < 0.05) that were consistently altered across both cell lines. Principal Component Analysis (PCA) and hierarchical clustering confirmed distinct transcriptomic profiles between control and knockdown groups (Fig. S3A–B). KEGG pathway enrichment analysis indicated that FZD3 depletion primarily perturbs metabolic networks—including nitrogen metabolism, arginine biosynthesis, and ether lipid metabolism—rather than canonical Wnt targets (Fig. S3C). Collectively, these data support the conclusion that FZD3 orchestrates SCLC aggressiveness via Wnt-independent metabolic and survival signaling axes.

## Discussion

Through the integration of multi-omics data, this study successfully identified FZD3 as a pivotal hub gene associated with SCLC, offering a more comprehensive understanding of the disease’s underlying mechanisms. Differential expression analysis between normal and disease groups revealed 1192 DEGs, comprising 534 upregulated and 658 downregulated genes. Subsequent application of two machine learning algorithms—LASSO regression and random forest—for screening potential hub genes, followed by intersection of their results, yielded three candidate core genes. Further multivariate risk model analysis identified FZD3 as a high-risk factor, leading to its selection as the central hub gene. The combination of machine learning and multivariate risk assessment enhanced the reliability of hub gene identification in SCLC. Our findings bridging bioinformatic prediction with experimental validation provide a novel perspective on SCLC progression.

Transcriptomic profiling revealed that FZD3-associated signatures are heavily enriched in immune-related and oncogenic signaling pathways, specifically leukocyte migration, cytokine-receptor interactions, and MAPK signaling. While inflammation is a general hallmark of cancer, the specific enrichment of pathways overlapping with HTLV-1 infection and NF-κB signaling suggests a profound dysregulation of immune surveillance and cell survival mechanisms in SCLC [30–34]. The MAPK pathway, a canonical regulator of proliferation and differentiation, is frequently co-opted in neuroendocrine tumors. Our data imply that FZD3 may act as an upstream modulator of these networks, orchestrating a tumor microenvironment conducive to metastasis [35–38]. The clinical stratification analysis underscores the prognostic value of FZD3. Elevated FZD3 expression was strictly correlated with advanced clinical stage and poor overall survival, positioning it as a potential stratification tool for high-risk patients [39].

To corroborate our bioinformatic predictions, we performed functional in vitro assays, confirming that FZD3 knockdown significantly abrogates the aggressive phenotypes of SCLC cells—specifically proliferation, migration, and invasion—while triggering apoptosis. However, the signaling dependency of FZD3 in SCLC appears distinct from its role in other malignancies. In the majority of solid tumors, FZD3 acts as a canonical activator of the Wnt/β-catenin axis. For instance, in breast cancer, osteosarcoma, and glioma, FZD3 is modulated by non-coding RNAs (e.g., 5’-tiRNAVal, lncRNA SNHG10, miR-139) to sustain β-catenin-driven metastasis and stemness [40–42]. Similarly, in colorectal cancer, FZD3 is obligatory for Wnt pathway activation downstream of genetic variants or HOXC10 [43, 44]. In stark contrast, our results indicate that FZD3 drives SCLC progression independently of this canonical axis, pointing to a lineage-specific mechanism.

The Wnt-independent mechanism we observed echoes the role of FZD3 in neural development. FZD3, acting via non-canonical Wnt/PCP signaling, is essential for neural progenitor fate and wiring [45, 46]. Given the neuroendocrine origin of SCLC, it is plausible that FZD3 hijacks these conserved developmental programs to drive tumor plasticity and invasiveness. This “neural mimicry” may also explain the aggressive metastatic tropism of SCLC. Our observation of Wnt-independent activity aligns with emerging evidence in melanoma, where FZD3 drives metastasis independent of β-catenin/TCF engagement [47]. Structurally, the ability of FZD3 to adopt active conformations without Wnt ligands—a feature revealed by recent cryo-EM studies—supports our finding of intrinsic, ligand-independent activity in SCLC [48]. Although the structural flexibility of the FZD3 cysteine-rich domain (CRD) poses druggability challenges, the unique absence of the H8 helix in the FZD3/6 subfamily offers a specific vulnerability for next-generation biologics, such as nanobodies and PROTACs [49–51].

Combining FZD3 inhibitors with conventional chemotherapy in the future holds promise for establishing a novel therapeutic model that breaks through drug resistance and improves clinical efficacy. Research evidence indicates that FZD3 expression positively correlates with the multidrug resistance gene MDR1. Suppressing its expression can restore sensitivity to drugs like cisplatin and doxorubicin by downregulating efflux pump activity [52]. Furthermore, considering the role of the related receptor FZD6 in DNA repair, targeting FZD3 may induce synthetic lethality when combined with DNA-damaging agents [53]. Furthermore, given FZD3’s critical role in sustaining cancer stem cells (CSCs) and tumor initiation in aggressive malignancies like melanoma, its inhibition may effectively eliminate the drug-resistant microenvironment driving post-treatment recurrence in lung cancer [54, 55]. This multifaceted synergistic effect—combining reversal of multidrug resistance, inhibition of DNA repair, and elimination of cancer stem cells—positions FZD3-targeted combination therapy as a high-potential strategy for future precision oncology.

## Conclusion

In conclusion, our study demonstrates that FZD3 is upregulated in SCLC and is associated with poor prognosis, indicating its role as a key driver in SCLC pathogenesis. Furthermore, FZD3 significantly influences the biological behaviors of SCLC cells, promoting proliferation, migration, and invasion. Notably, knockdown of FZD3 induced SCLC cell apoptosis, suggesting that FZD3 may represent a promising therapeutic target in SCLC. Future studies based on combination treatment of FZD3 targeting and chemo/radio-based therapies or immunotherapy in SCLC will strengthen its foundation for clinical translation.

## Supplementary Information

Supplementary Material 1.

Supplementary Material 2.

Supplementary Material 3.

Supplementary Material 4.

Supplementary Material 5.

## Data Availability

The data that support the findings of this study are available on request from the corresponding author upon reasonable request.

## Abbreviations

SCLC: Small cell lung cancer
NSCLC: Non-small cell lung cancer
PCAWG: Pan-Cancer Analysis of Whole Genomes
TCGA: The Cancer Genome Atlas
PCA: Principal component analysis
GO: Gene Ontology
KEGG: Kyoto Encyclopedia of Genes and Genomes
CC: Cellular Component
BP: Biological Process
MF: Molecular Function
LASSO: Least Absolute Shrinkage and Selection Operator
HR: Hazard ratios
CI: Confidence intervals
GSVA: Gene set variation analysis
DEGs: Differentially expressed genes
TME: Tumor microenvironment
HE: Hematoxylin and eosin
mIHC: Multiplex immunohistochemical
CRP: C-reactive protein
HTLV-1: Human T-cell leukemia virus 1
PGs: Proteoglycans
GAGs: Glycosaminoglycans
ECM: Extracellular matrix
NPC: Neural progenitor cell
