# Supplementary material for: The Wnt receptor Frizzled3 (FZD3) drives aggressive phenotypes in small cell lung cancer
Source: Respir Res. 2026 Mar 21;27:192. doi: 10.1186/s12931-026-03634-1 (PMC13126896; doi:10.1186/s12931-026-03634-1)
Supplement: Supplementary file 1 — Supplementary Material 1. [file 12931_2026_3634_MOESM1_ESM.docx]

**Table S1.** The complete list of differentially expressed genes between the normal control group and the tumor group.

**Table S2.** Demographic and Clinical Information of SCLC Patients


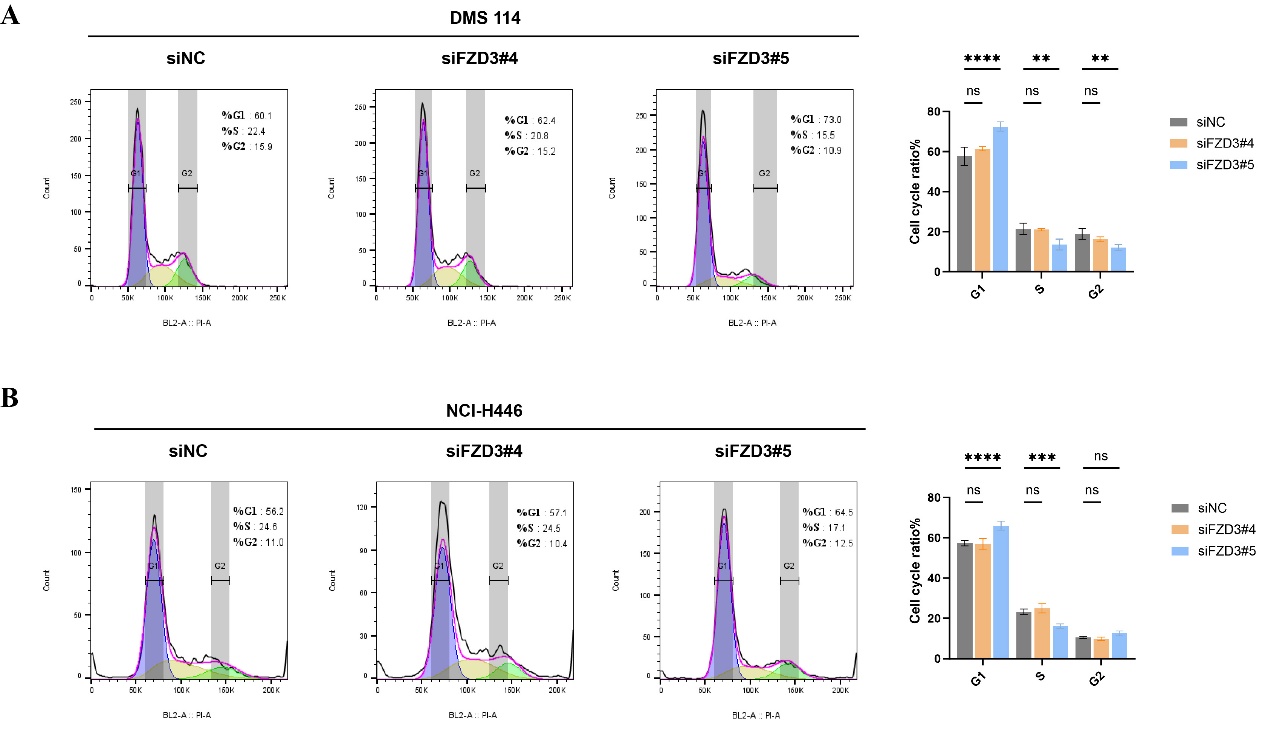


**Figure S1. Effect of FZD3 knockdown on cell cycle distribution in SCLC cells.**
**A and B.** Cell cycle profiles of SCLC cells after FZD3 knockdown analyzed by flow cytometry. One-way ANOVA analysis was performed. Error bars represent the standard deviation (SD) from three replicate experiments. * p < 0.05 **p < 0.01， *** p < 0.001， **** p < 0.0001


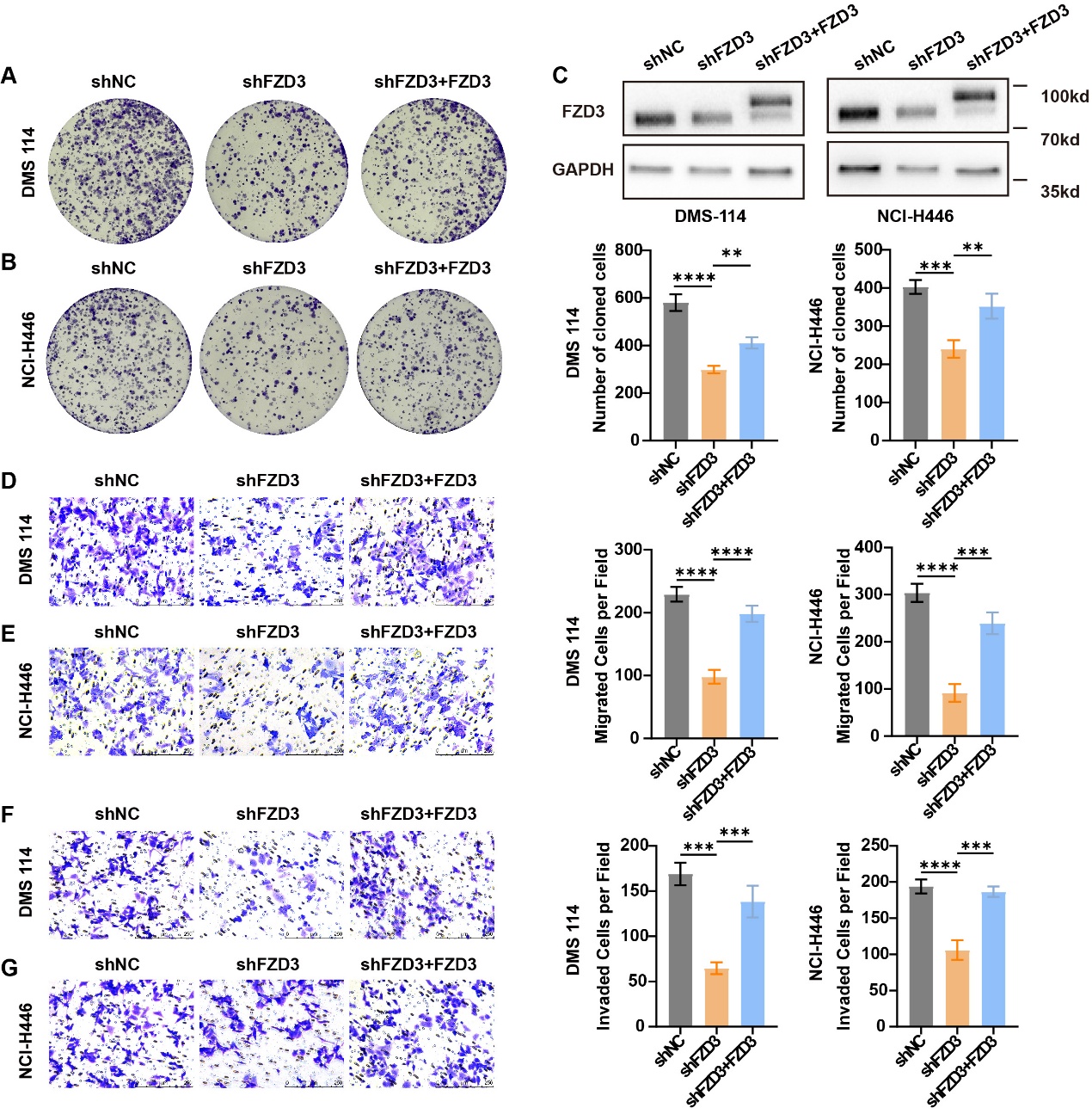


**Figure S2. Changes in Proliferation, Migration, and Invasion of SCLC Cells Following FZD3 Knockdown and Rescue.
A and B.** Detection of colony-forming capacity in FZD3-knockdown and rescued small cell lung cancer cells via colony formation assay (14 days). One-way ANOVA analysis was performed. Error bars represent the standard deviation (SD) from three replicate experiments.

**C.** Western blot experiments validated changes in FZD3 knockdown and rescue protein levels.

**D and E.** Migration ability of SCLC cells after FZD3 knockdown and rescue evaluated by Transwell assay. One-way ANOVA analysis was performed. Error bars represent the standard deviation (SD) from three replicate experiments. One-way ANOVA analysis was performed. Error bars represent the standard deviation (SD) from three replicate experiments.

**F and G.** Invasion ability of SCLC cells after FZD3 knockdown and rescue evaluated by Transwell assay. One-way ANOVA analysis was performed. Error bars represent the standard deviation (SD) from three replicate experiments. One-way ANOVA analysis was performed. Error bars represent the standard deviation (SD) from three replicate experiments. * p < 0.05 **p < 0.01， *** p < 0.001， **** p < 0.0001


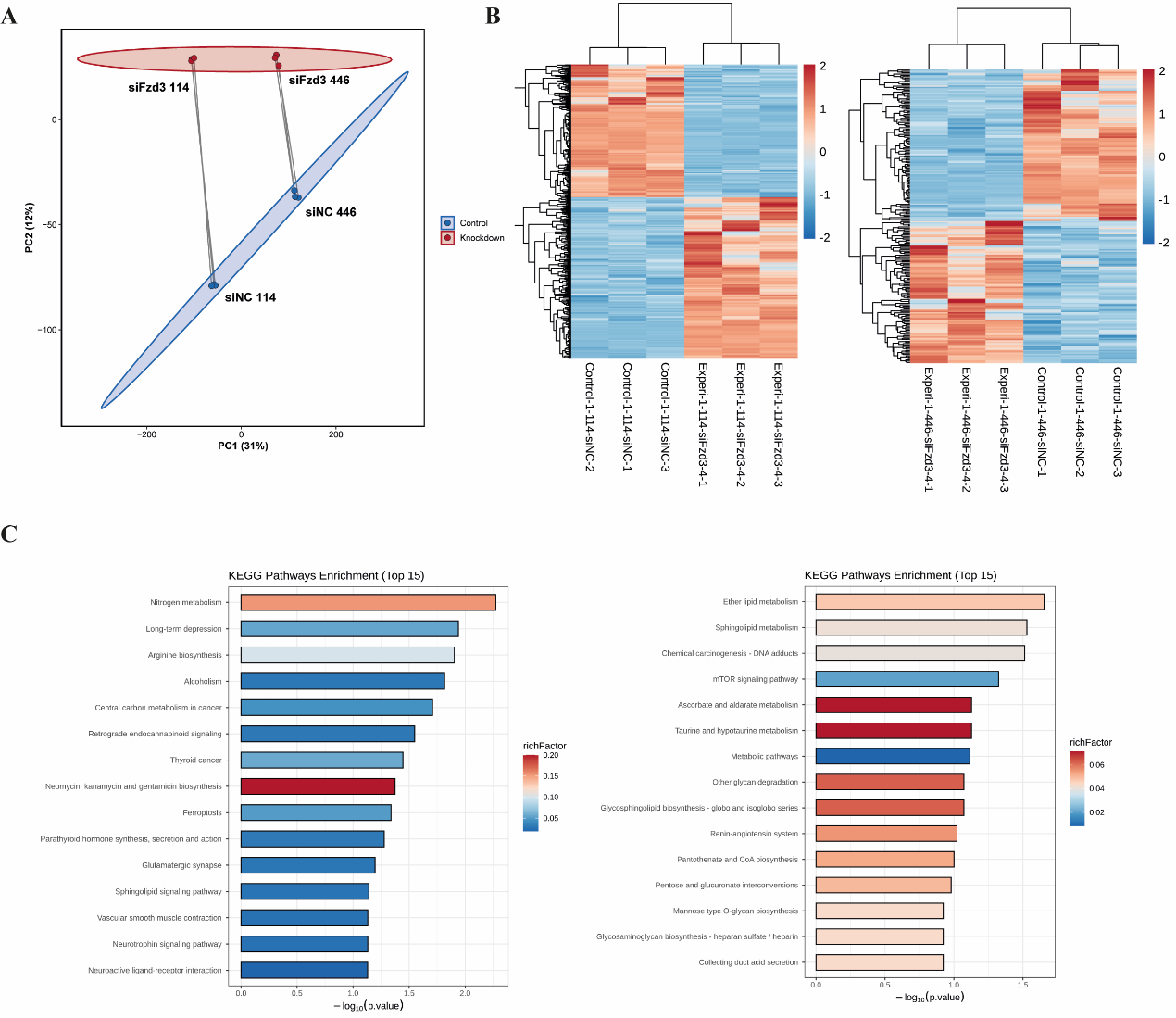


**Figure S3. Transcriptomic alterations induced by FZD3 knockdown in SCLC cells.**

**A.** Principal component analysis (PCA) of gene expression profiles in SCLC cells transfected with control siRNA (siNC) or FZD3-targeting siRNA (siFzd3) in two cell lines (DMS 114 and NCI-H446)

**B.** Heatmaps showing hierarchical clustering of differentially expressed genes (DEGs) in 114 cells (left) and 446 cells (right) after FZD3 silencing compared with controls

**C.** KEGG pathway enrichment analysis (top 15 pathways) of DEGs in 114 cells (left) and 446 cells (right) following FZD3 knockdown.
