# Supplementary material for: The Wnt receptor Frizzled3 (FZD3) drives aggressive phenotypes in small cell lung cancer
Source: Respir Res. 2026 Mar 21;27:192. doi: 10.1186/s12931-026-03634-1 (PMC13126896; doi:10.1186/s12931-026-03634-1)
Supplement: Supplementary file 4 — Supplementary Material 4. [file 12931_2026_3634_MOESM4_ESM.pptx]

## Slide 1
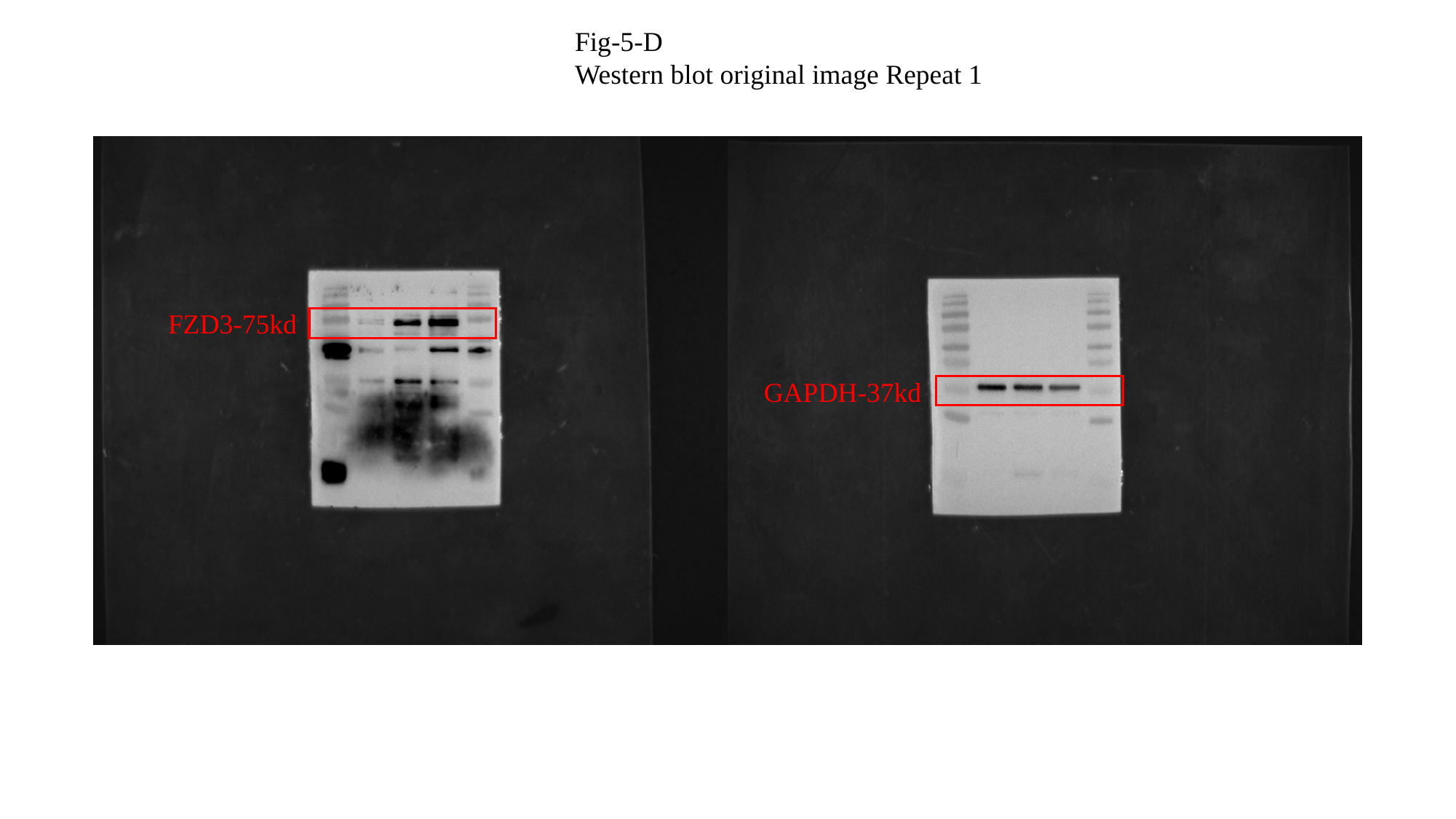

Fig-5-D
Western blot original image Repeat 1
FZD3-75kd
GAPDH-37kd

## Slide 2
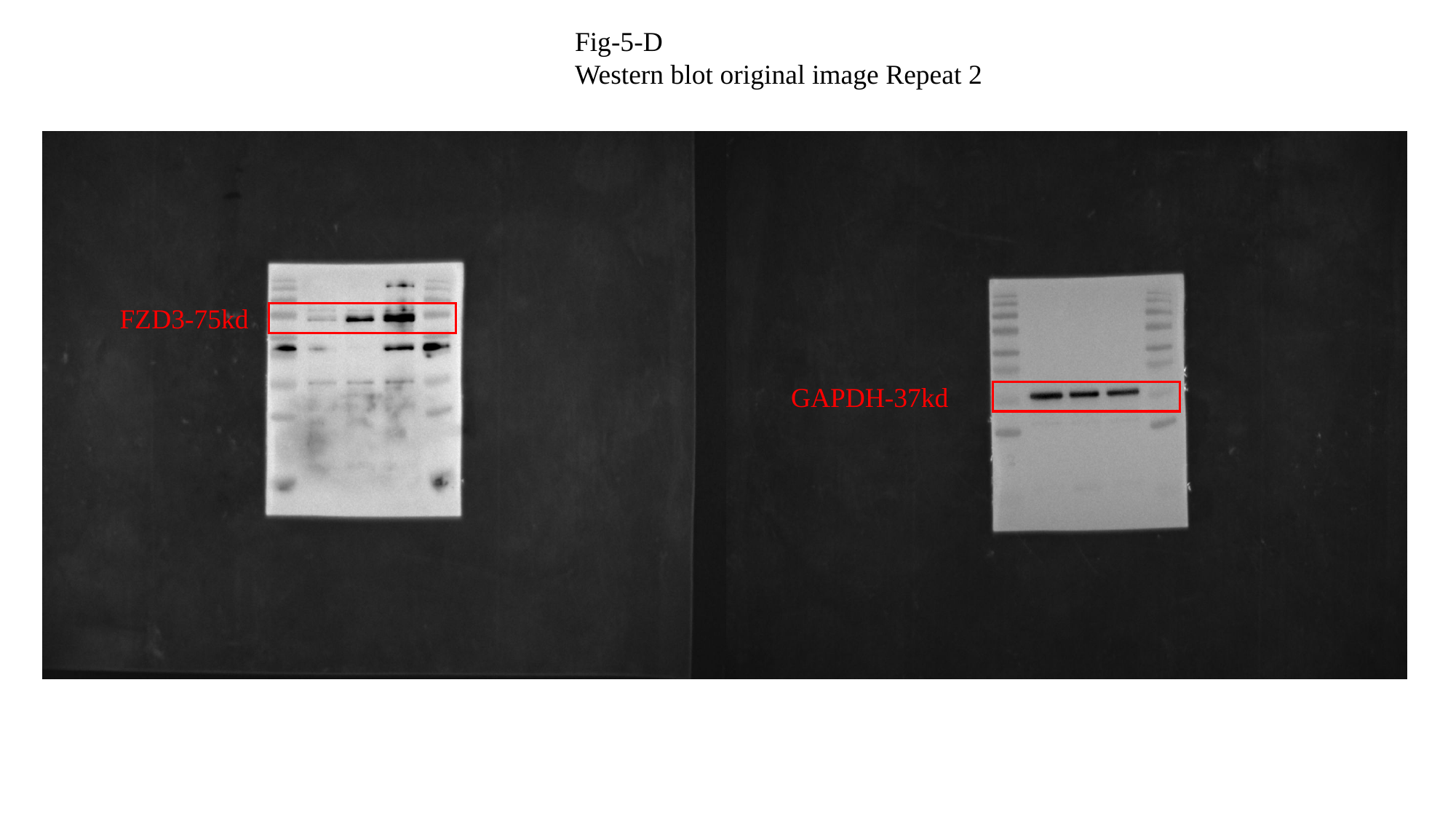

Fig-5-D
Western blot original image Repeat 2
FZD3-75kd
GAPDH-37kd

## Slide 3
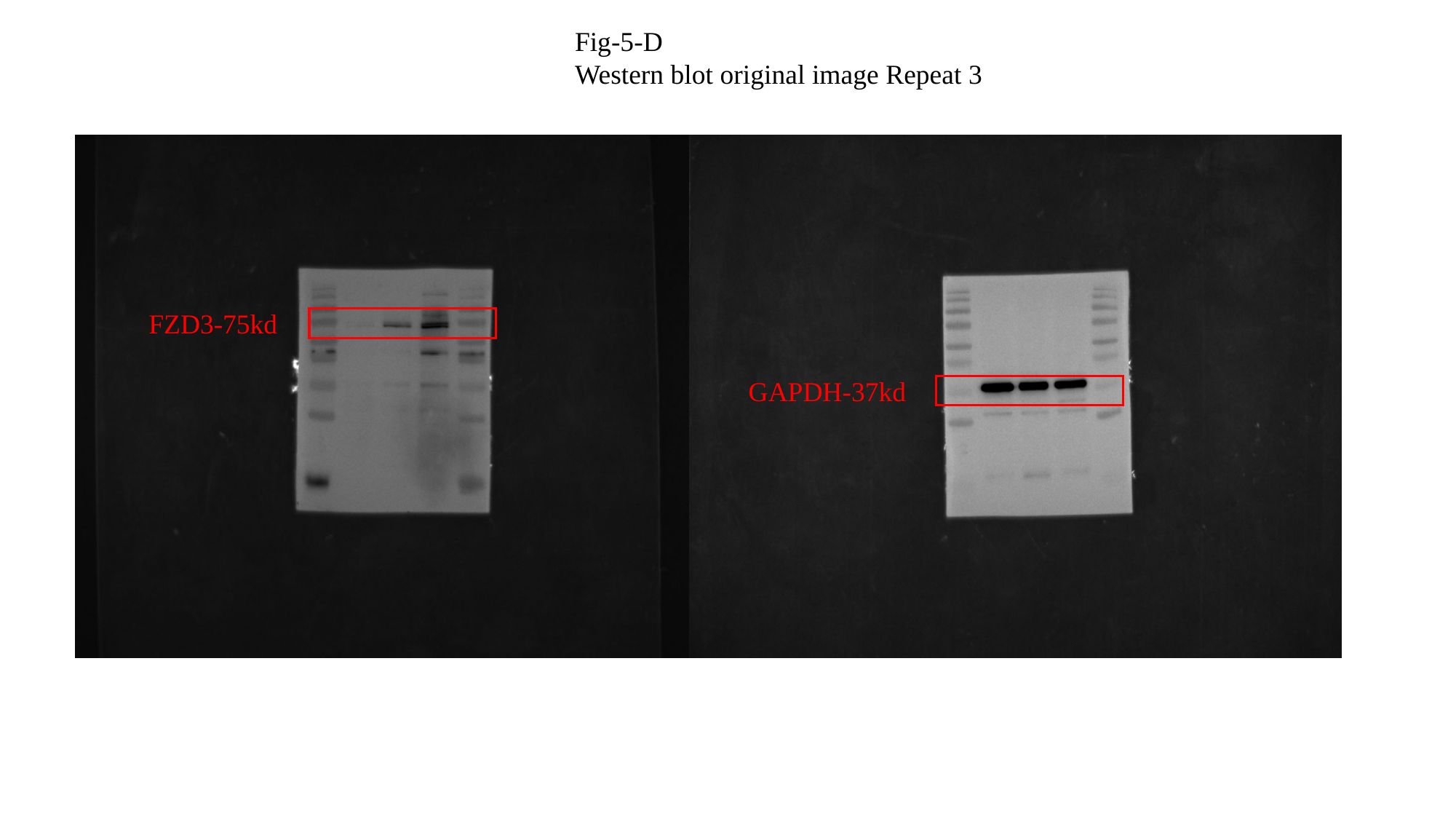

Fig-5-D
Western blot original image Repeat 3
FZD3-75kd
GAPDH-37kd

## Slide 4
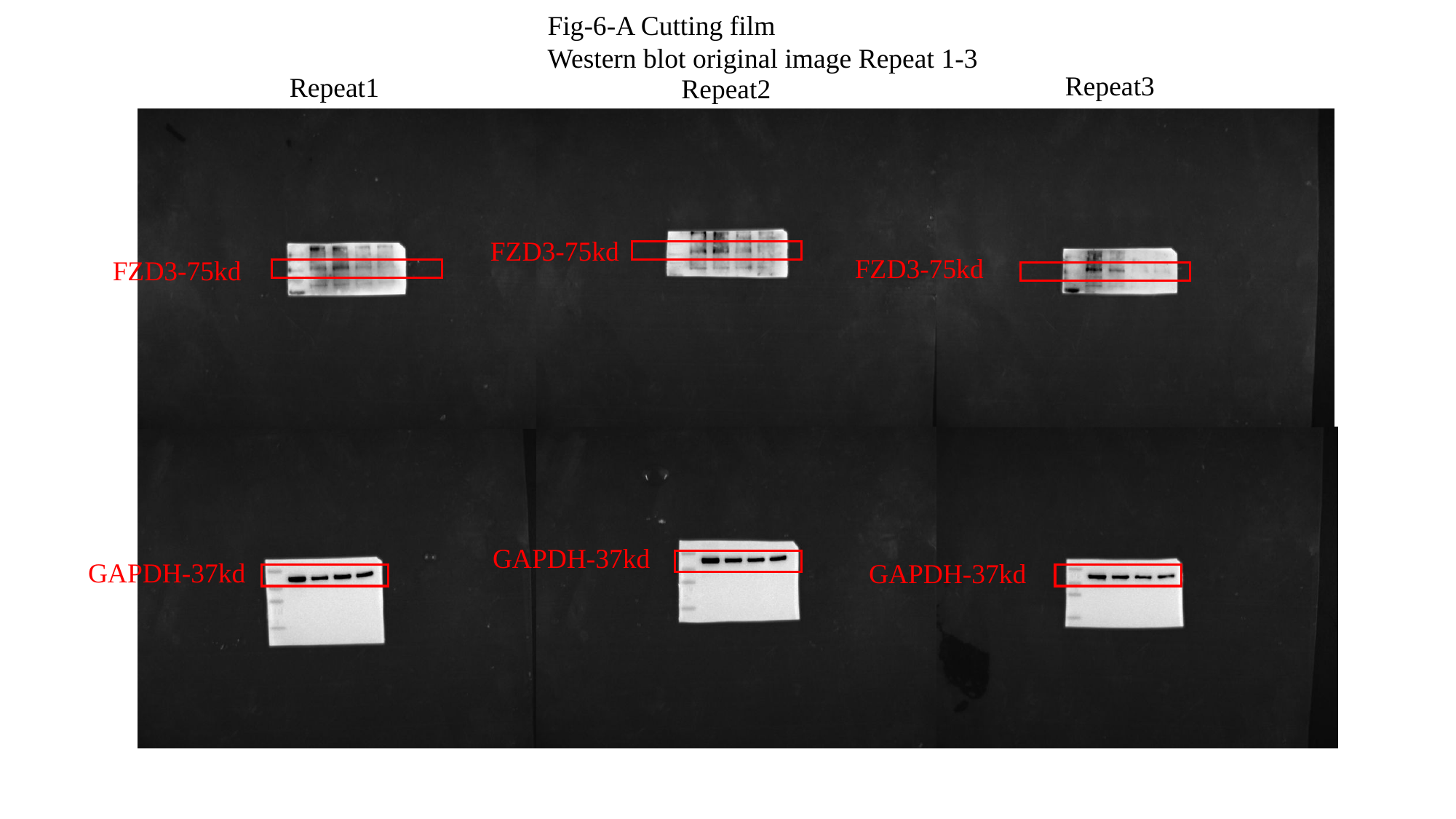

Fig-6-A Cutting film
Western blot original image Repeat 1-3
Repeat3
Repeat1
Repeat2
FZD3-75kd
FZD3-75kd
FZD3-75kd
GAPDH-37kd
GAPDH-37kd
GAPDH-37kd

## Slide 5
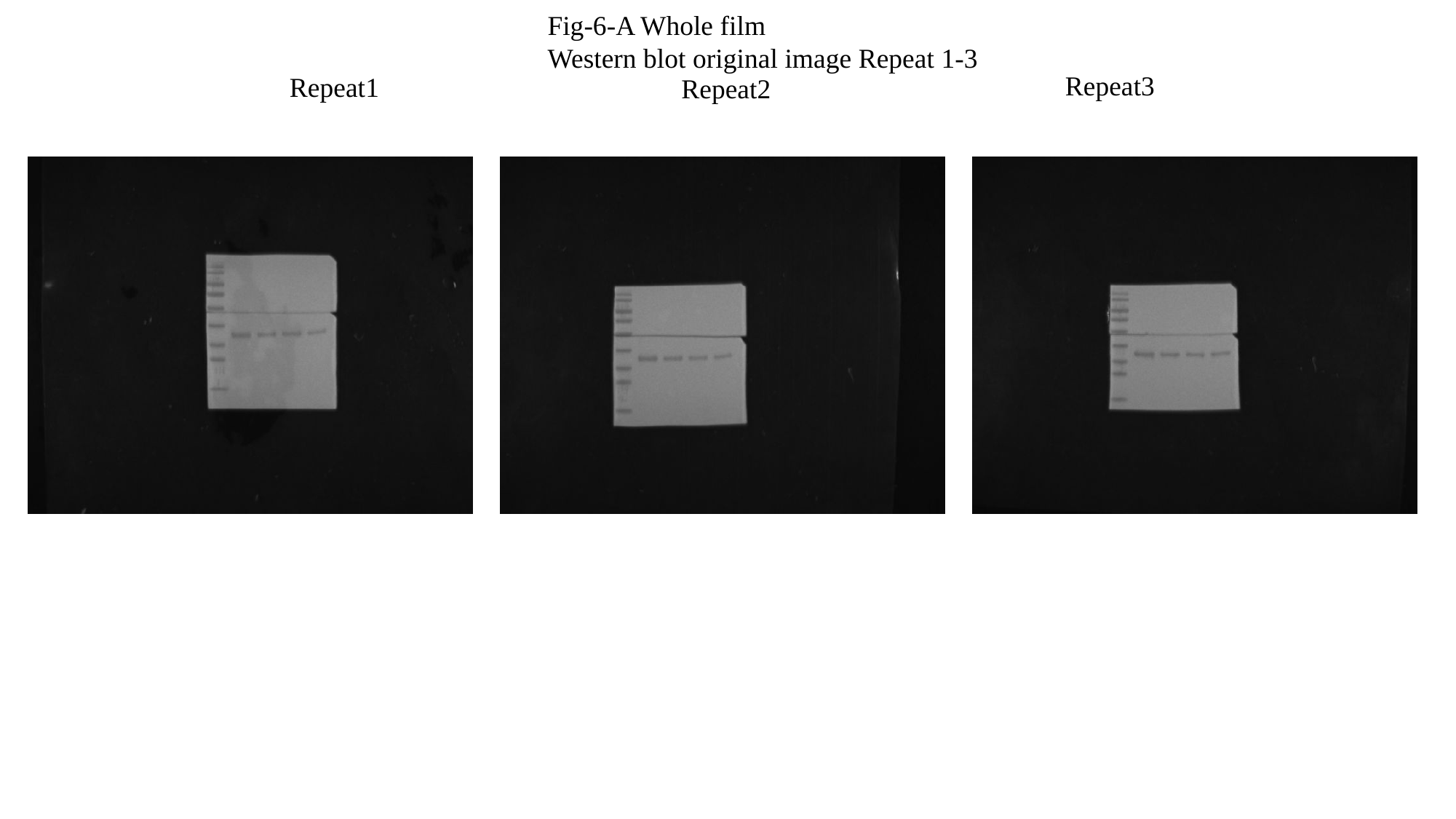

Fig-6-A Whole film
Western blot original image Repeat 1-3
Repeat3
Repeat1
Repeat2

## Slide 6
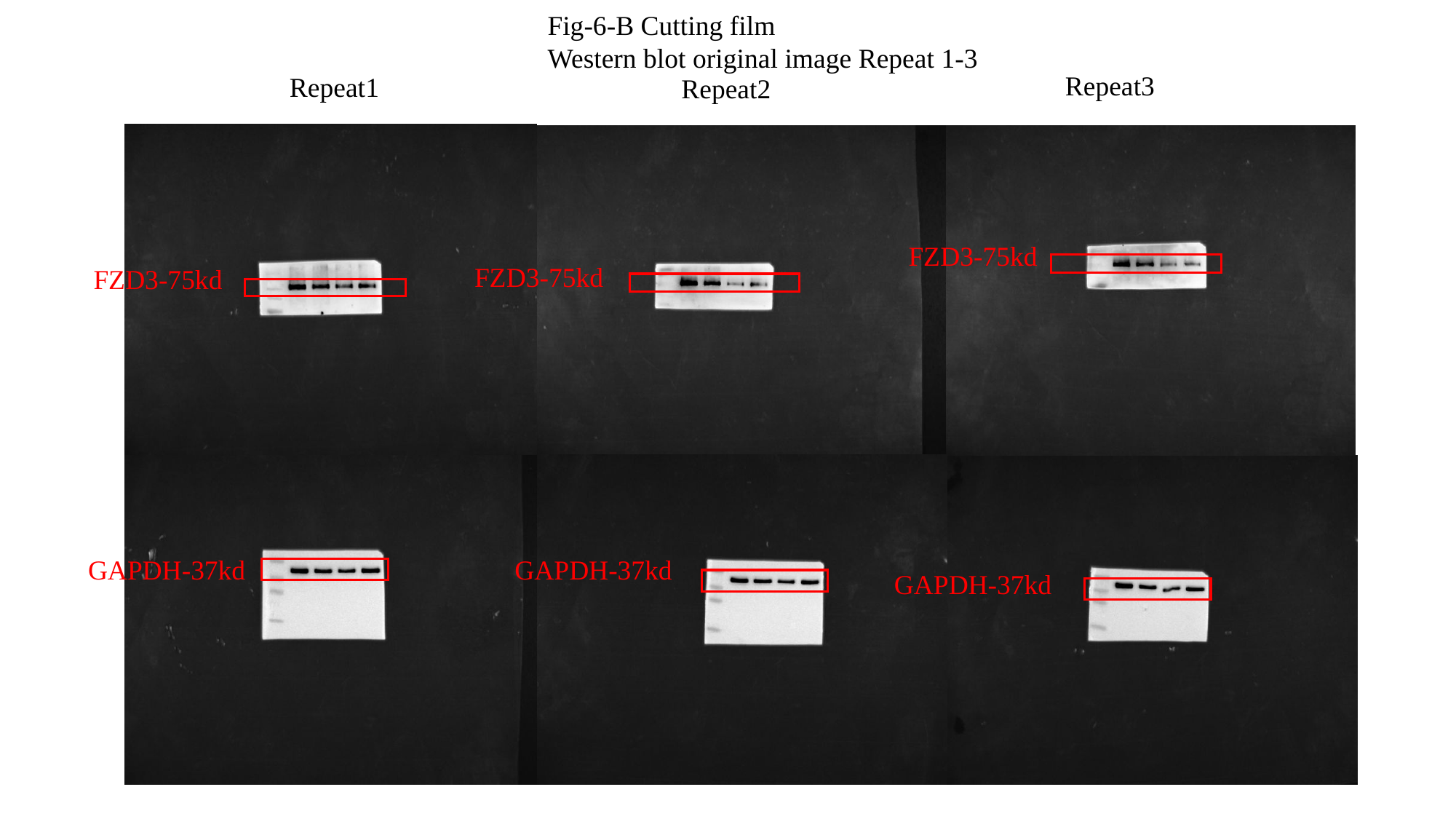

Fig-6-B Cutting film
Western blot original image Repeat 1-3
Repeat3
Repeat1
Repeat2
FZD3-75kd
FZD3-75kd
FZD3-75kd
GAPDH-37kd
GAPDH-37kd
GAPDH-37kd

## Slide 7
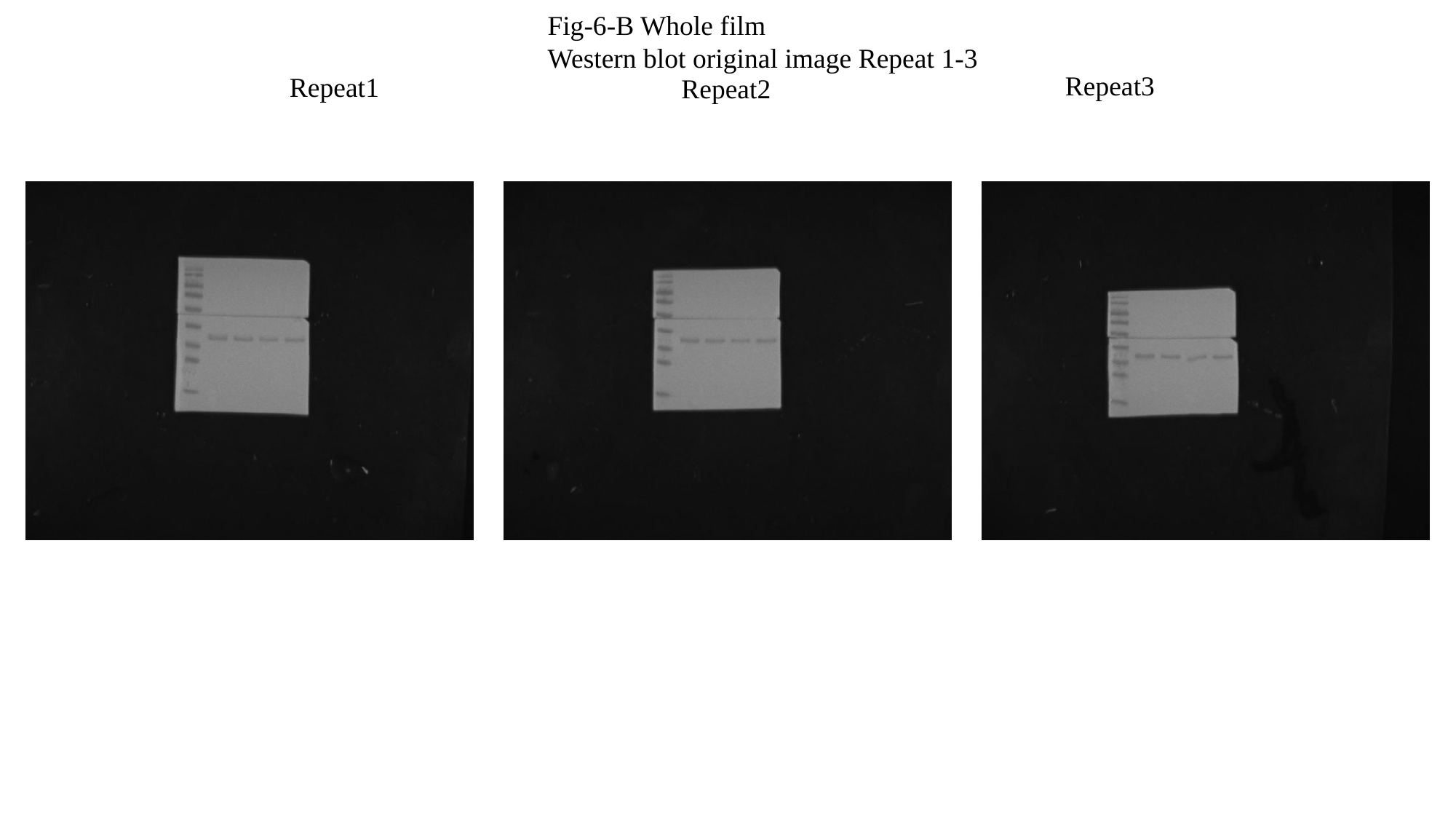

Fig-6-B Whole film
Western blot original image Repeat 1-3
Repeat3
Repeat1
Repeat2

## Slide 8
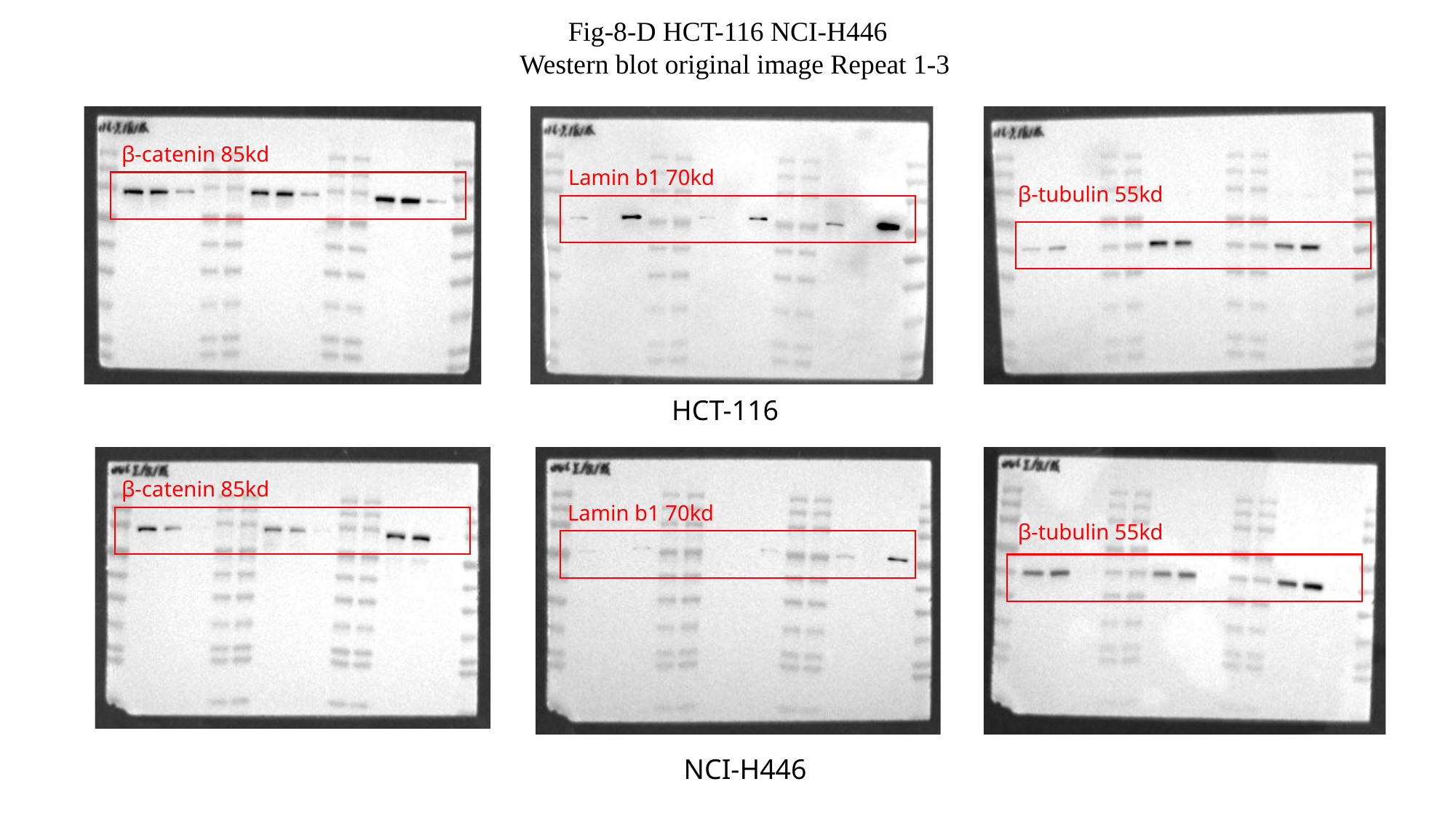

Fig-8-D HCT-116 NCI-H446
Western blot original image Repeat 1-3
β-catenin 85kd
Lamin b1 70kd
β-tubulin 55kd
HCT-116
β-catenin 85kd
Lamin b1 70kd
β-tubulin 55kd
NCI-H446

## Slide 9
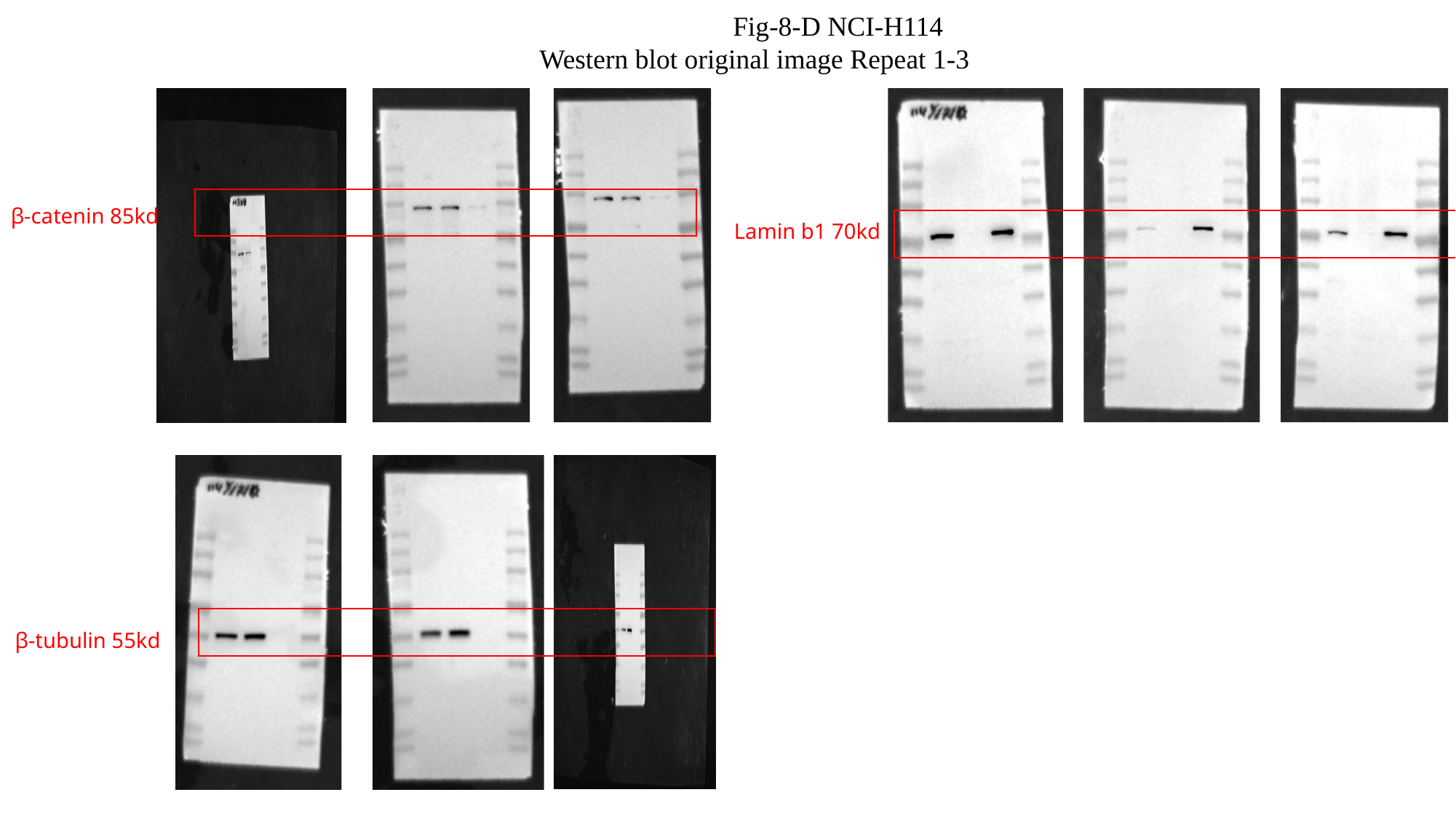

Fig-8-D NCI-H114
Western blot original image Repeat 1-3
β-catenin 85kd
Lamin b1 70kd
β-tubulin 55kd

## Slide 10
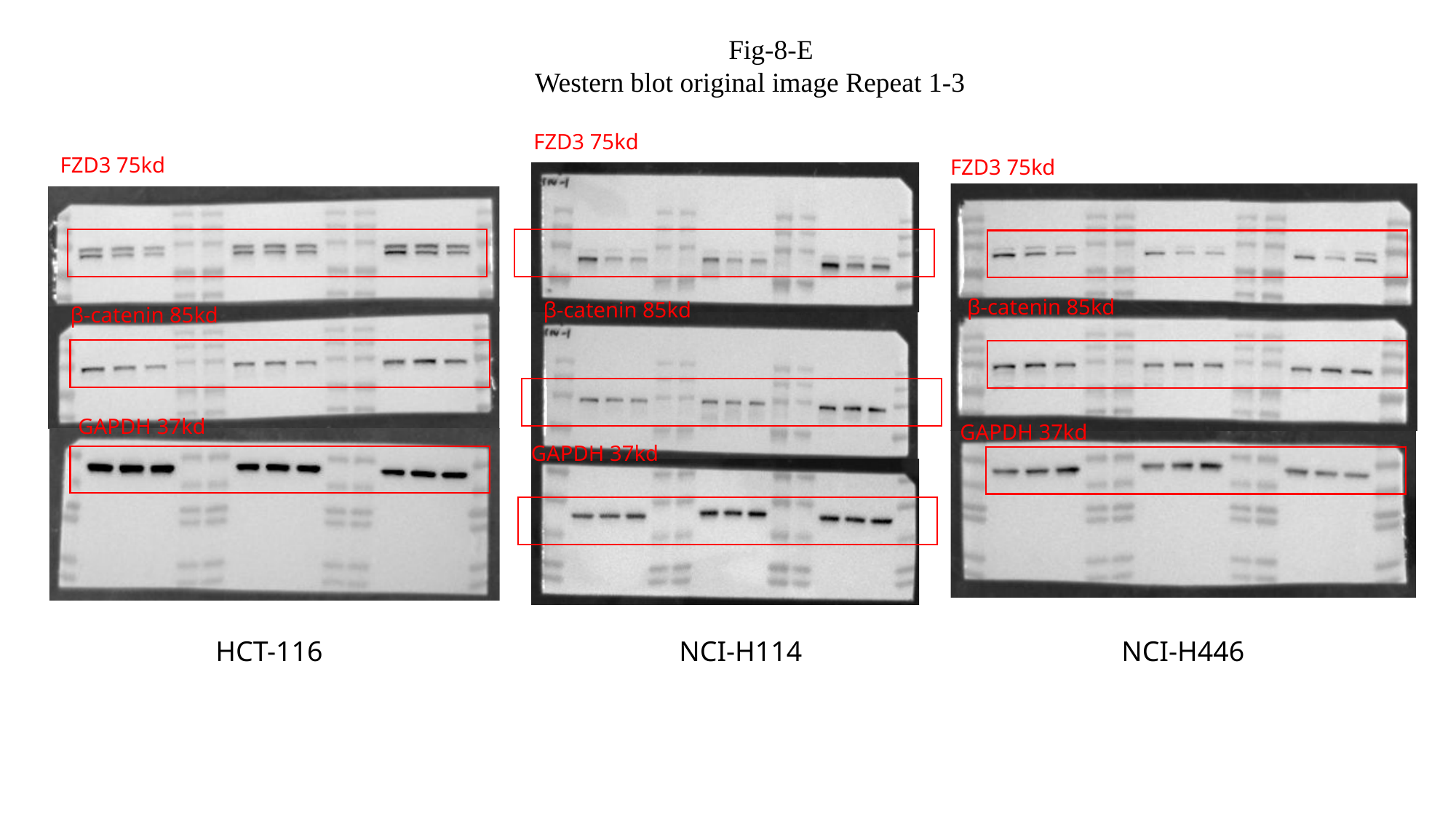

Fig-8-E
Western blot original image Repeat 1-3
FZD3 75kd
FZD3 75kd
FZD3 75kd
β-catenin 85kd
β-catenin 85kd
β-catenin 85kd
GAPDH 37kd
GAPDH 37kd
GAPDH 37kd
HCT-116
NCI-H114
NCI-H446

## Slide 11
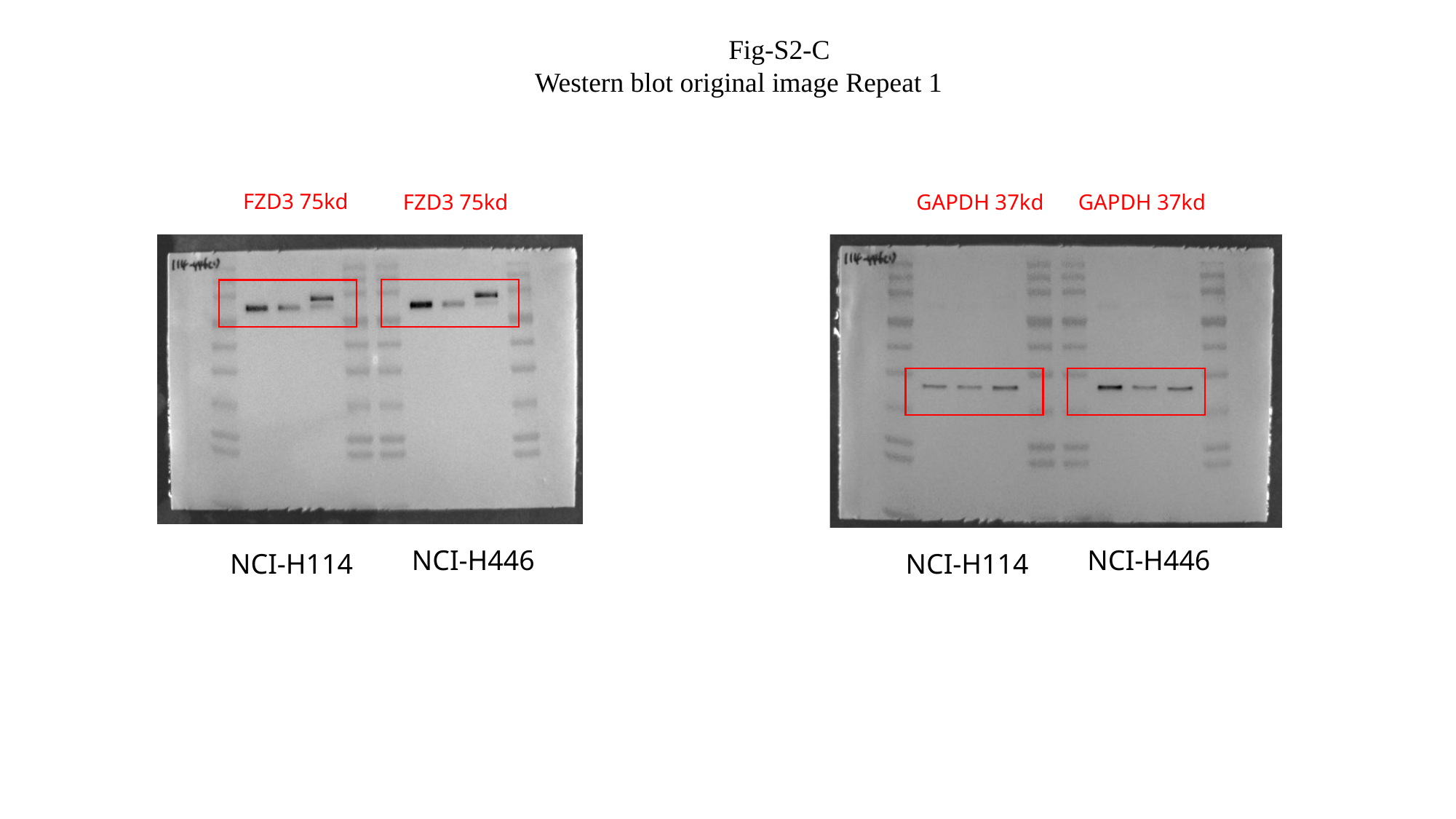

Fig-S2-C
Western blot original image Repeat 1
FZD3 75kd
FZD3 75kd
GAPDH 37kd
GAPDH 37kd
NCI-H446
NCI-H446
NCI-H114
NCI-H114

## Slide 12
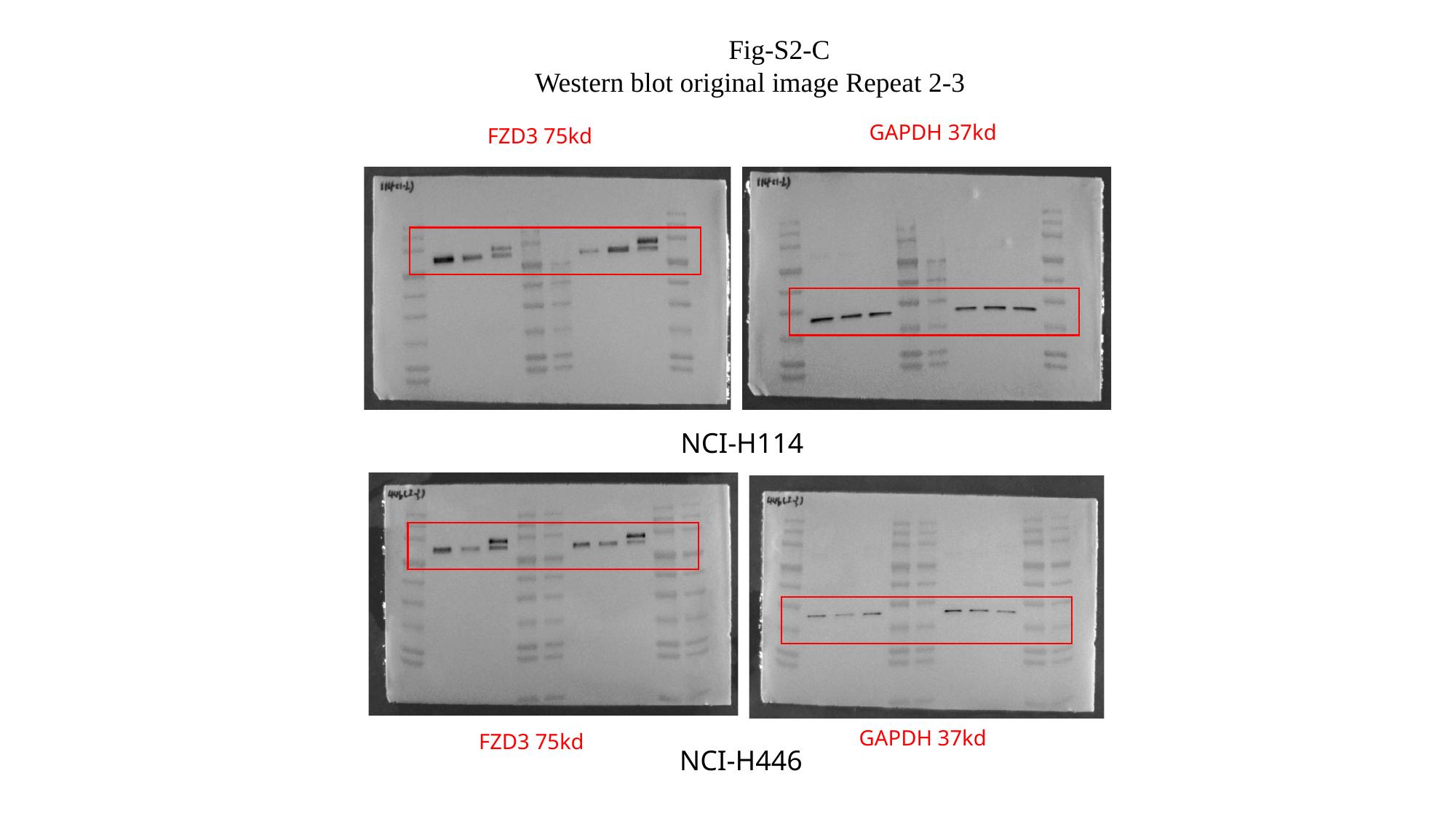

Fig-S2-C
Western blot original image Repeat 2-3
GAPDH 37kd
FZD3 75kd
NCI-H114
GAPDH 37kd
FZD3 75kd
NCI-H446
