# Supplementary figures and images for: The Wnt receptor Frizzled3 (FZD3) drives aggressive phenotypes in small cell lung cancer
Source: Respir Res. 2026 Mar 21;27:192. doi: 10.1186/s12931-026-03634-1 (PMC13126896; doi:10.1186/s12931-026-03634-1)

Ethics Approval Number: LW-2024-022


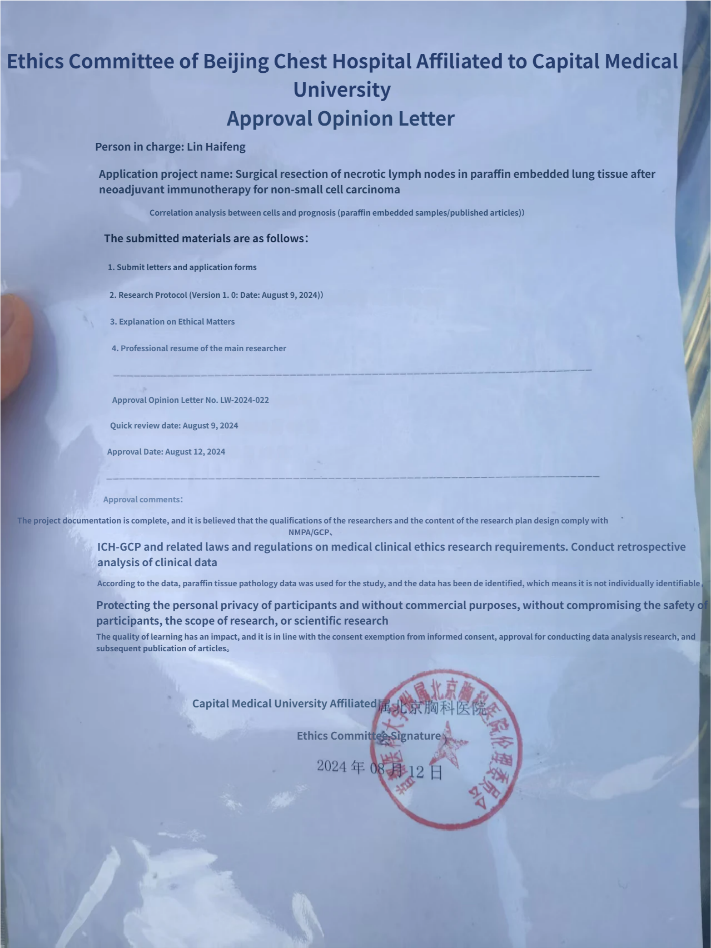

Supplement: Supplementary file 5 — Supplementary Material 5. [file 12931_2026_3634_MOESM5_ESM.docx]
